# Mn vacancies in Mn_3_O_4_: steering lithiation kinetics during solid-state synthesis toward ultrastable spinel cathode materials

**DOI:** 10.1039/d6sc06403e

**Published:** 2026-09-23

**Authors:** Rui-Xiang Wang, Yu-Qi Zhou, Jia-Qi Huang, Ce-Heng Duan, Jia-Jie Zhang, Le-Yi Zhong, Zhi-Zhong Shen, Xue-Chun Huang, Bin He, Xiao-Juan Chen, Tian-Yuan Xiao, Dan Xiao, Yan Meng

**Affiliations:** a School of Chemical Engineering, Institute of New Energy and Low-Carbon Technology, Sichuan University Chengdu 610065 Sichuan China mengyan@scu.edu.cn

## Abstract

The lithiation process during solid-state synthesis governs lattice formation, defect evolution and, ultimately, the electrochemical performance of lithium-ion battery cathodes. However, how the competition between lithiation and oxidation during precursor conversion dictates the formation of the final crystal lattice remains poorly understood. Here we reveal the decisive role of competitive lithiation in regulating lattice formation and defect evolution during the conversion of a commercially relevant Mn_3_O_4_ precursor into spinel LiMn_2_O_4_ (LMO), and establish the underlying thermodynamic and kinetic mechanisms. We show that an Mn_3_O_4_ precursor containing intrinsic Mn defects exhibits a lower lithiation formation energy, enabling rapid and homogeneous early-stage lithiation that suppresses the formation of the Mn_2_O_3_ intermediate and eliminates competitive lithiation. This pathway promotes the direct reconstruction of the Mn–O framework, yielding LMO with higher lattice integrity, a lower oxygen-vacancy concentration and a more stable Mn–O coordination environment. Consequently, the material delivers an initial discharge capacity of 135.19 mAh g^−1^ at 5C and retains 90.13% of its capacity after 500 cycles. Rather than relying on conventional post-synthesis optimization strategies such as elemental doping or surface modification, this work establishes a precursor-regulated lithiation strategy that provides new mechanistic insights and general design principles for the controllable synthesis of Mn_3_O_4_-derived manganese-based cathode materials.

## Abstract 2

Mn vacancies in Mn_3_O_4_ precursors accelerate lithiation and suppress Mn_2_O_3_ formation, enabling more uniform Mn–O framework reconstruction and producing LiMn_2_O_4_ with improved lattice integrity and Li^+^ transport.

## Graphical abstract

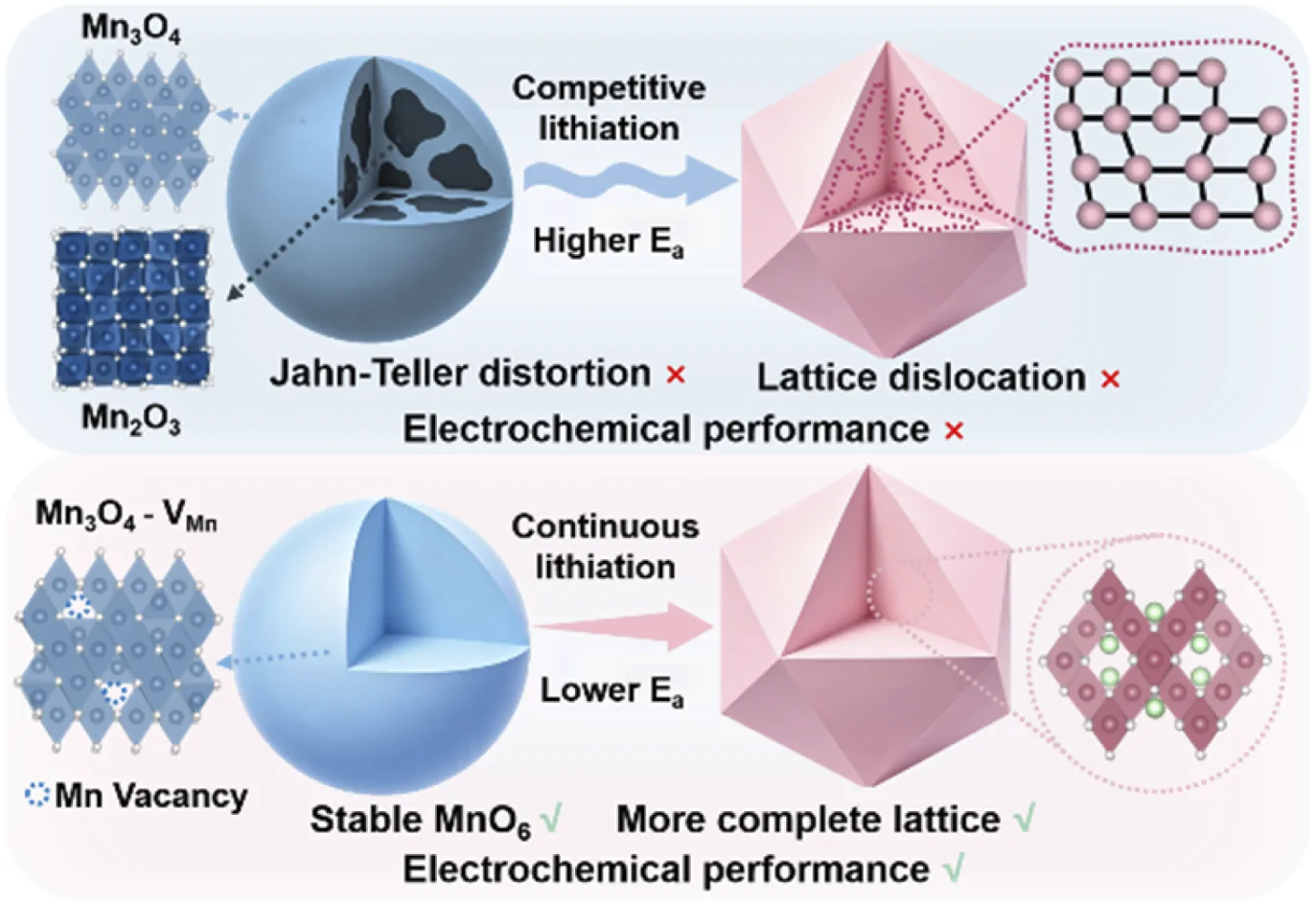

## Introduction

With the growing demand for ultrafast charge–discharge capability in high-power lithium-ion batteries, spinel LiMn_2_O_4_ (LMO), which features a three-dimensional Li^+^ transport network, has regained considerable attention as an attractive cathode material.^1–4^ However, the practical electrochemical performance of LMO remains far below the fast ion-transport capability anticipated from its ideal crystal structure.^5,6^ Moreover, a perfectly stoichiometric and structurally ideal LMO lattice is difficult to obtain under practical synthesis conditions.^7^ This discrepancy primarily arises from the thermodynamic and kinetic mismatch among multiple elementary reactions involved in conventional solid-state synthesis. In particular, the decomposition of lithium sources, oxidation of Mn-based precursors, Li^+^ diffusion, reconstruction of the spinel lattice and oxygen exchange do not necessarily proceed synchronously, and are often governed by distinct reaction environments.^8–10^ When the intrinsic structure of the precursor is not properly controlled, insufficient lithiation, local deficiency and reaction heterogeneity can be introduced, thereby promoting the formation of Jahn–Teller-active high-spin Mn^3+^.^11^ The resulting distortion of MnO_6_ octahedra accelerates lattice-strain accumulation, weakens the stability of the Mn–O framework and triggers structural degradation during repeated charge–discharge cycling, ultimately leading to poor cycling stability.^12^ In addition, oxygen defects, cation disorder and local lattice distortions in non-ideal LMO structures can disrupt the continuity of the Li^+^ migration network and increase local diffusion barriers, thus limiting reversible capacity and rate capability.^13^ Therefore, the unsatisfactory performance of LMO essentially stems from the coupled evolution of precursor-induced defect formation, key lithiation pathways, local lattice reconstruction and Mn valence states during synthesis. In recent years, result-oriented regulation of the structure and interface of LMO has been extensively explored, including optimization of the Li/Mn ratio, ion doping and surface coating.^14–18^ These strategies have been demonstrated to partially suppress Jahn–Teller distortion, mitigate Mn dissolution and improve interfacial stability. Nevertheless, most previous studies have focused on the final structural modification and electrochemical outcomes, whereas the essential processes involved in the precursor-to-spinel transformation, including lithiation reactions, oxygen exchange, Mn valence evolution, lattice rearrangement and defect formation, remain insufficiently understood. In fact, precursor-to-spinel transformation is not a simple high-temperature crystallization process, but a multistep reaction governed jointly by redox thermodynamics and ion-transport kinetics. A systematic understanding of the thermodynamic and kinetic behaviour throughout the lithiation process is therefore crucial for precisely regulating the Mn^3+^/Mn^4+^ ratio, suppressing lattice distortion and structural defects, and stabilizing the Mn–O framework.

Early phase-diagram studies have shown that the Li/Mn stoichiometry, compositional stability window and oxygen non-stoichiometry of the spinel phase are closely associated with temperature and oxygen partial pressure.^19,20^ This indicates that the sintering atmosphere and nucleation temperature not only determine phase formation, but also directly affect the Mn^3+^/Mn^4+^ ratio, oxygen-defect concentration and local structural ordering. In recent years, increasing evidence has further demonstrated that the structural characteristics of cathode materials are not dictated solely by equilibrium phase stability, but are strongly regulated by precursor-conversion pathways, lithiation barriers and phase-evolution processes.^21–24^ For example, constructing abundant Ni–O–Mn configurations on the precursor surface has been shown to accelerate charge-transfer kinetics and lower the Li^+^ insertion barrier, thereby suppressing nanopore formation in Ni-rich cathode materials.^25^ In addition, ultrafast thermal shock-induced dehydration of FeSO_4_·7H_2_O precursors can facilitate the decomposition of the Na_2_Fe(SO_4_)_2_ intermediate and promote the formation of phase-pure alluaudite-type sodium iron sulfate.^26^ Collectively, these studies highlight that understanding the thermodynamic driving forces and kinetic limitations during cathode sintering has become an important route towards precise crystal-structure regulation. For LMO, different Mn-based precursors differ markedly in structural openness, Mn–O coordination environment, redox activity and resistance to Li^+^ diffusion; therefore, their lithiation behaviours and spinel-lattice formation pathways can be substantially different. Previous studies have shown that the lithiation kinetics can be controlled through precursor-dependent structural transformation pathways, enabling the homogeneous synthesis of Li-excess LMO.^27^ It has also been reported that the formation of certain impurity phases during sintering can significantly influence the morphological evolution of LMO crystals.^28^ These findings suggest that clarifying the structural evolution mechanism during the transformation from Mn-based precursors to spinel LMO is essential for regulating the Mn^3+^/Mn^4+^ ratio, suppressing lattice-defect formation and stabilizing the Mn–O framework from the earliest stage of synthesis.

**Fig. 1:**
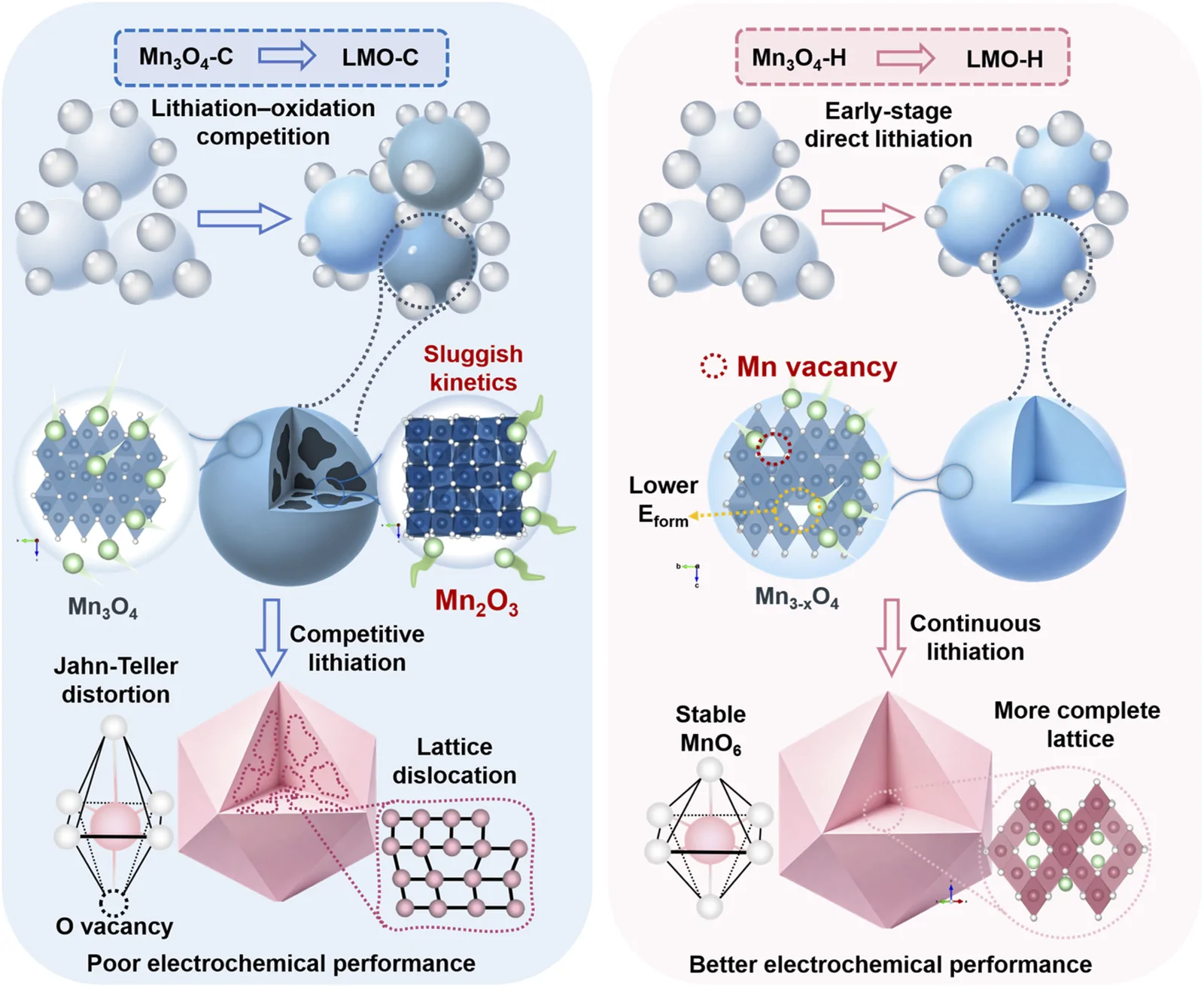
Schematic illustration of the distinct lithiation reaction pathways from Mn_3_O_4_ to LMO and their effects on the structure of the resulting products.

Motivated by these considerations, a precursor widely adopted in industrial production (Mn_3_O_4_), as a model system to elucidate how lithiation pathways govern LMO lattice formation, defect evolution and electrochemical performance. Specifically, the transformation from Mn_3_O_4_ to LMO is controlled by the competition between lithiation and oxidation. Rapid and homogeneous early-stage lithiation promotes the direct reconstruction of the Mn–O framework, leading to a highly ordered spinel lattice with enhanced structural integrity and superior electrochemical performance. In contrast, delayed lithiation introduces an additional phase-reconstruction process, giving rise to lattice defects that deteriorate structural stability and electrochemical performance (Fig. 1). We further demonstrate that the intrinsic lattice characteristics of the precursor also regulate the thermodynamics of lithiation, thereby fundamentally influencing spinel-lattice formation and the properties of the final product. Rather than relying on conventional strategies based on elemental doping or surface modification, this work establishes a precursor-regulated synthesis strategy centred on controlling the early-stage lithiation process. These mechanistic insights provide an effective route for constructing LMO cathodes with high lattice integrity, robust structural stability and rapid Li^+^ transport, and offer broader guidance for the controllable preparation of other Mn_3_O_4_-derived manganese-based cathodes, including Li-rich layered oxides and spinel lithium nickel manganese oxide.

## Experimental

### Material synthesis

Preparation of the Mn_3_O_4_-H precursor: a 2 M MnSO_4_·H_2_O (99.9%, Aladdin) solution and a 5 M NaOH (≥98%, Greagent) solution were first prepared. The MnSO_4_ solution was used as the starting solution, and the NaOH solution was added dropwise under continuous stirring. The amount of NaOH added was adjusted to maintain the final pH at 11. After the addition was completed, the suspension was continuously stirred for 3 h to ensure complete precipitation. The resulting precipitate was collected by filtration, repeatedly washed with deionized water, and dried overnight at 120 °C to obtain the Mn_3_O_4_-H precursor.

Preparation of the Mn_3_O_4_-H-800 precursor: the as-prepared Mn_3_O_4_-H precursor was calcined at 800 °C for 3 h under an O_2_ atmosphere to obtain the Mn_3_O_4_-H-800 precursor.

Preparation of the Mn_3_O_4_-C-Bead Milling precursor: commercial Mn_3_O_4_-C powder was dispersed in ethanol (or deionized water) under continuous stirring to form a homogeneous suspension. The suspension was then subjected to bead milling for 6 h, followed by spray drying to obtain the Mn_3_O_4_-C-Bead Milling precursor.

Preparation of LiMn_2_O_4_: LMO-H, LMO-C and LMO-C-Bead Milling were synthesized using the same solid-state reaction procedure. Prior to lithiation, the Mn contents of Mn_3_O_4_-H and Mn_3_O_4_-C were quantitatively determined by inductively coupled plasma optical emission spectroscopy (ICP-OES) (SI Table 1). Based on the measured Mn contents, the amount of Li_2_CO_3_ was calculated according to the stoichiometric Li/Mn molar ratio of 1 : 2, with an additional 2% excess Li_2_CO_3_ introduced to compensate for possible Li loss during high-temperature calcination. The corresponding Mn_3_O_4_ precursor was thoroughly mixed with the calculated amount of Li_2_CO_3_. Then the mixtures were ball-milled at 400 rpm for 4 h with a ball-to-powder mass ratio of 10 : 1 using zirconia balls. The mixture was subsequently calcined at 800 °C for 6 h under an O_2_ atmosphere to obtain the desired LiMn_2_O_4_ products. For the preparation of LMO-H-800, equal masses of the Mn_3_O_4_-H precursor was first pre-calcined at 800 °C for 3 h under an O_2_ atmosphere. After cooling to room temperature, the pre-calcined powder was ball-milled with the corresponding amount of Li_2_CO_3_, determined according to the Mn content and the same lithiation protocol described above, and subsequently calcined again at 800 °C for 6 h under the same O_2_ atmosphere to obtain the LMO-H-800 sample. The final Li/Mn ratios of LMO-H, LMO-H-800, LMO-C, and LMO-C-Bead Milling were further quantified by ICP-OES and were all found to be close to the designed stoichiometric ratio (SI Table 2), confirming that the differences in their electrochemical performance are not associated with appreciable variations in the final Li/Mn composition.

### Material characterization

XRD patterns were collected over a 2*θ* range of 10–80° using an X-ray diffractometer (SmartLab SE, Rigaku, Japan) equipped with Cu Kα radiation (*λ* = 1.5406 Å). Rietveld refinements were performed using the FullProf software package to obtain detailed crystallographic information. For *in situ* high-temperature XRD measurements, the Mn_3_O_4_ precursor and Li_2_CO_3_ were mixed in the stoichiometric ratio required for LiMn_2_O_4_ formation, loaded onto a heating stage and heated at a rate of 5 °C min^−1^. Diffraction patterns were recorded at 20 °C intervals over a 2*θ* range of 10–80° at a scanning rate of 10° min^−1^. The morphology of the samples was examined by SEM (SU8200, Hitachi, Japan), whereas their morphology and microstructure were further characterized by TEM and HR-TEM (JEM-F200, JEOL, Japan) operated at an accelerating voltage of 200 kV. Raman spectra were collected using a Raman spectrometer (inVia Qontor, Renishaw, UK) with a 532 nm excitation laser after the powder samples had been gently flattened onto quartz coverslips. Thermogravimetric analysis was performed using a simultaneous thermal analyser (TGA/DSC 3+, Mettler Toledo, Switzerland), for which the Mn_3_O_4_ precursor and Li_2_CO_3_ were mixed in the prescribed stoichiometric ratio and heated under an oxygen atmosphere. The surface chemical states of the samples were investigated by XPS (K-Alpha, Thermo Scientific, USA), with the binding energies calibrated against the C 1s peak at 284.6 eV. Oxygen-vacancy-related paramagnetic species were examined by EPR (EMXplus-6/1, Bruker, Germany). The local chemical environment of Li was investigated by solid-state nuclear magnetic resonance spectroscopy (MAS20, HUPRISING, China). XAFS measurements were conducted at room temperature at the BL17B1 beamline of the National Facility for Protein Science in Shanghai at the Shanghai Synchrotron Radiation Facility. The amount of Mn deposited on the Li-metal anodes after electrochemical cycling was quantified by ICP-OES (5100 SVDV, Agilent, USA). CV and EIS measurements were conducted using a dual-channel electrochemical workstation (PARSTAT 3000A-DX, Princeton Applied Research, USA). Further details of the characterization procedures are provided in the SI.

## Results and discussion

### Structural characterization

All diffraction peaks of the synthesized precursor (Mn_3_O_4_-H) and commercial precursor (Mn_3_O_4_-C) were indexed to the Mn_3_O_4_ phase, and no reflections attributable to impurity phases were detected, as confirmed by X-ray diffraction (XRD) (Fig. 2a and S1). Upon high-temperature lithiation, Mn_3_O_4_-H and Mn_3_O_4_-C were converted into the corresponding LiMn_2_O_4_ products, hereafter denoted LMO-H and LMO-C, respectively. Both products crystallized in the cubic spinel structure with the *Fd*3̄*m* space group, without any discernible impurity phases. (Fig. 2b and c). Compared with LMO-C, LMO-H displays sharper and better-defined diffraction peaks, indicating its higher crystallinity. Further Rietveld refinement shows that LMO-H possesses a larger lattice parameter, which may provide more open Li^+^ diffusion channels, thereby favouring rapid Li^+^ transport (SI Table 3). Distinct local environments of the MnO_6_ octahedra are also observed in different samples. The A_1g_ stretching vibration band of LMO-H shifts towards a higher wavenumber, suggesting strengthened Mn–O bonding and enhanced stability of the MnO_6_ octahedral framework. In addition, the increased intensity of the F_2g_ band, observed as a weak shoulder at approximately 598 cm^−1^, indicates a higher average Mn valence state in LMO-H (Fig. 2d). This higher Mn valence is expected to reduce the fraction of Mn^3+^ and suppress Jahn–Teller distortion.^29^

**Fig. 2:**
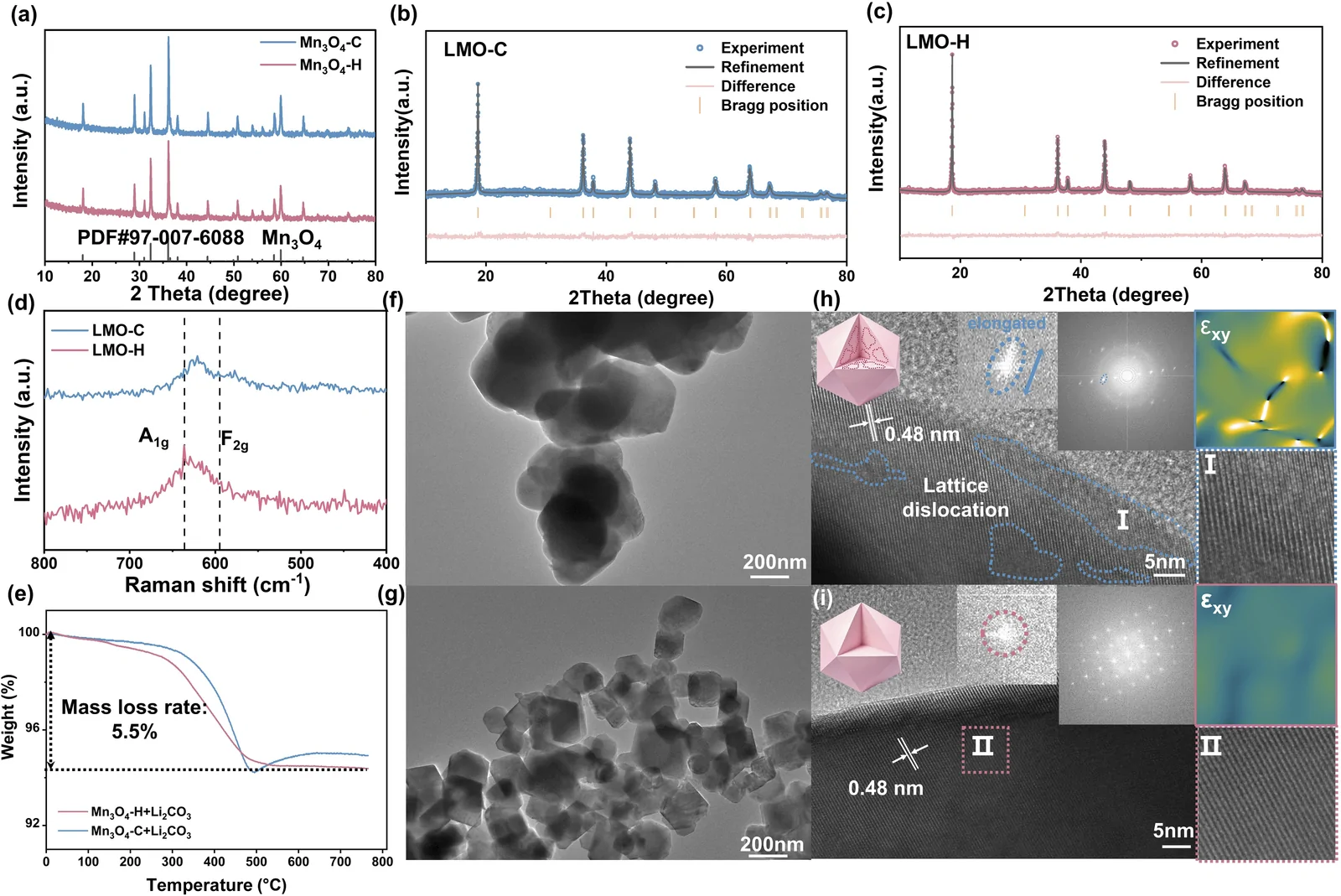
Structural and morphological characterization of the Mn_3_O_4_ precursors and LMO products. (a) XRD patterns of Mn_3_O_4_-H and Mn_3_O_4_-C. (b and c) Rietveld refinement profiles of (b) LMO-C and (c) LMO-H. (d) Raman spectra of LMO-C and LMO-H. (e) Thermogravimetric profiles of the reactions between Li_2_CO_3_ and Mn_3_O_4_-H or Mn_3_O_4_-C. (f and g) TEM images of (f) LMO-C and (g) LMO-H. (h) HR-TEM image of LMO-C, together with an enlarged view of the lattice-dislocated region and the corresponding geometrical phase analysis map. (i) HR-TEM image of LMO-H, together with an enlarged lattice-resolved image and the corresponding geometrical phase analysis map.

The different lithiation kinetics of the two Mn_3_O_4_ precursors are further reflected in their thermogravimetric behaviours (Fig. 2e). Compared with the Mn_3_O_4_-C/Li_2_CO_3_ system, the Mn_3_O_4_-H/Li_2_CO_3_ mixture undergoes obvious mass loss at a lower temperature, indicating that lithiation can be initiated earlier and proceeds with more favourable reaction kinetics. Notably, the Mn_3_O_4_-C/Li_2_CO_3_ system exhibits an inflection in mass change after approximately 500 °C, accompanied by a certain degree of mass increase. This behaviour suggests that a distinct reaction process occurs at the later stage of lithiation, which is likely associated with oxygen exchange and reconstruction of the spinel lattice.

The contrasting morphological evolution and crystallization behavior of the two precursors are further revealed by scanning electron microscopy (SEM) images at different sintering temperatures (Fig. S2). In the Mn_3_O_4_-H system, the particles exhibit more pronounced crystallization features within the temperature range of 200–400 °C, suggesting that structural rearrangement is activated at a relatively low temperature. By contrast, the Mn_3_O_4_-C system shows a clear enhancement in crystallinity only at approximately 600 °C, at which the initially well-defined particles gradually coalesce into polycrystalline-like aggregates with blurred grain boundaries. Such divergent crystal-growth behaviours ultimately result in distinct particle morphologies in the final LMO products. LMO-C mainly exhibits a polycrystalline-like morphology formed by the fusion of multiple primary particles, whereas LMO-H displays well-dispersed single-crystal-like particles with more complete crystal morphology (Fig. 2f and g).

At the lattice scale, LMO-C contains evident local dislocation structures, accompanied by elongated fast Fourier transform diffraction spots. To further clarify the origin of these dislocations, the lattice structure of an intermediate collected from the Mn_3_O_4_-C/Li_2_CO_3_ system during calcination at 600 °C was analysed. The results reveal that LiMn_2_O_4_ has already formed near the particle surface, whereas the particle core became as Mn_2_O_3_, with local lattice distortions and dislocation structures appearing in the transition region between these domains (Fig. S3 and S4). This spatially resolved structural evolution further supports that the dislocations are closely associated with local lattice mismatch arising from the distinct lithiation pathways (Fig. 2h and S5). The presence of dislocations can further aggravate lattice distortion and introduce stronger shear strain within the lattice, thereby weakening the structural stability during electrochemical cycling. In contrast, LMO-H shows higher crystallinity, continuous and well-ordered lattice fringes, and lower lattice microstrain, suggesting that it undergoes a more uniform and continuous lithiation process (Fig. 2i and S6). Notably, LMO-H-800 also exhibits relatively uniform lattice fringes without obvious extended dislocations, indicating that pronounced dislocation formation is not an inevitable consequence of lithiation through a single-phase precursor (Fig. S7). Nevertheless, the distinct lithiation pathways can still lead to differences in local lattice reconstruction, point-defect chemistry, and the Mn–O coordination environment, which may contribute to the inferior electrochemical performance of LMO-H-800 relative to LMO-H.

### Lithiation kinetics during sintering

To further elucidate the lithiation kinetics and phase-transformation pathways of different Mn_3_O_4_ precursors during sintering, the phase evolution during heating was tracked by *in situ* high-temperature XRD. Compared with the Mn_3_O_4_-C/Li_2_CO_3_ system, the Mn_3_O_4_-H/Li_2_CO_3_ system develops characteristic reflections of spinel LiMn_2_O_4_ (*Fd*3̄*m* space group) at a lower temperature, indicating that Mn_3_O_4_-H enters the effective lithiation stage earlier and exhibits faster lithiation kinetics (Fig. 3c). The formation of the spinel phase at a reduced temperature suggests that Li^+^ can participate in the reconstruction of the Mn–O framework more readily, thereby promoting the direct transformation of Mn_3_O_4_ into LMO.

**Fig. 3:**
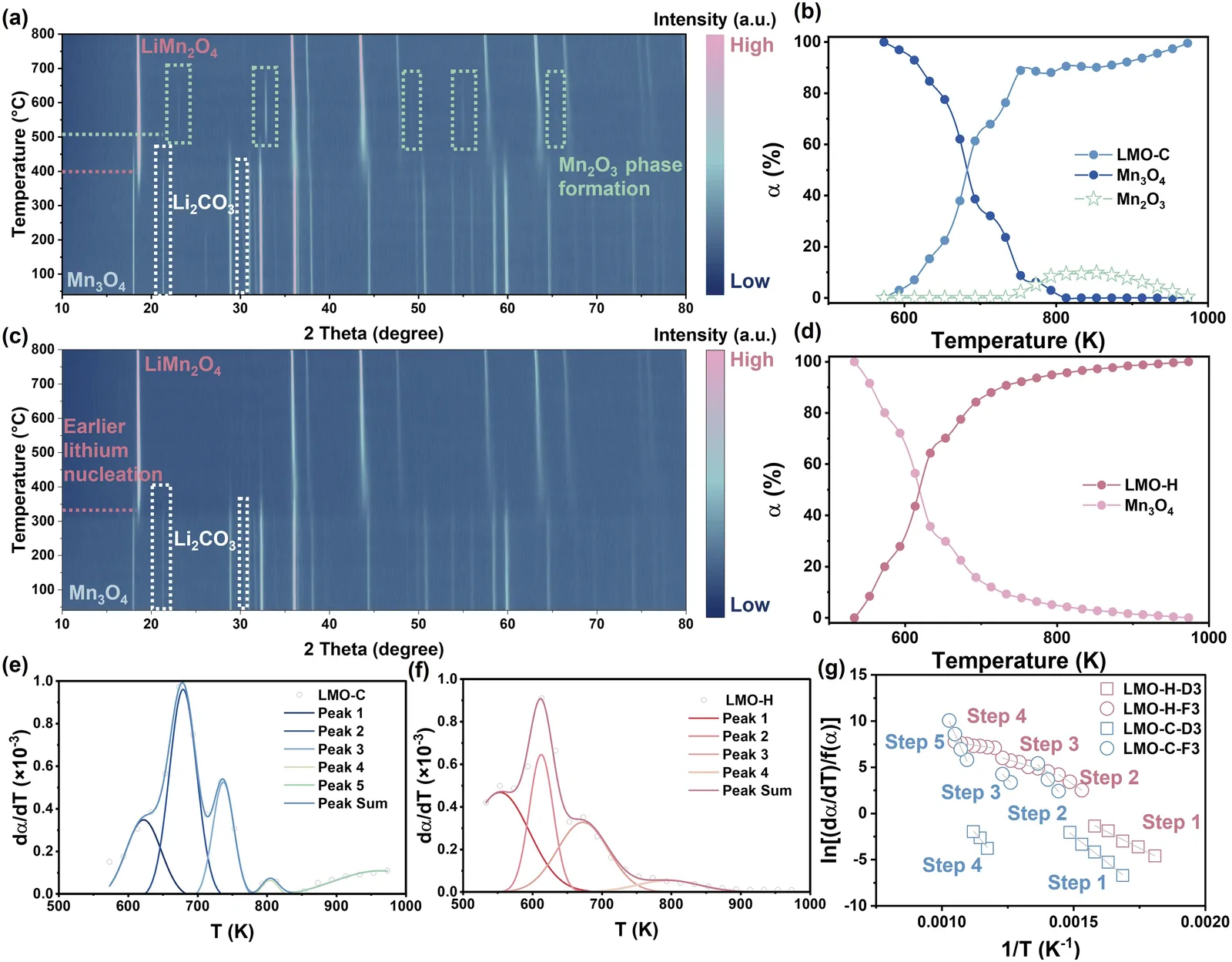
Precursor-dependent lithiation pathways and reaction kinetics during solid-state sintering. (a and c) *In situ* high-temperature XRD contour maps of the (a) Mn_3_O_4_-C/Li_2_CO_3_ and (c) Mn_3_O_4_-H/Li_2_CO_3_ mixtures. (b and d) Temperature-dependent phase-fraction evolution of the (b) Mn_3_O_4_-C/Li_2_CO_3_ and (d) Mn_3_O_4_-H/Li_2_CO_3_ systems, as derived from multiphase Rietveld refinement. (e and f) Temperature derivatives of the relative LMO fraction *α* for (e) LMO-C and (f) LMO-H. (g) Arrhenius plots for the formation of LMO-C and LMO-H.

By contrast, characteristic reflections of Mn_2_O_3_ emerge in the Mn_3_O_4_-C/Li_2_CO_3_ system after approximately 500 °C, suggesting that delayed lithiation allows Mn_3_O_4_ to undergo oxidative phase transformation before complete conversion to LMO (Fig. 3a). Therefore, the later stage of sintering in the Mn_3_O_4_-C system is not governed by a single lithiation process, but involves competing reactions among direct lithiation of Mn_3_O_4_, oxidation of Mn_3_O_4_ and subsequent lithiation of Mn_2_O_3_. Such a competitive phase-transformation pathway increases the complexity of the reaction process and may lead to local lithiation heterogeneity, non-uniform lattice reconstruction and accumulation of defect structures. The corresponding reaction processes can be described as follows:8 Mn_3_O_4_ + 6 Li_2_CO_3_ +5 O_2_ = 12 LiMn_2_O_4_ + 6 CO_2_4 Mn_3_O_4_ + O_2_ = 6 Mn_2_O_3_4 Mn_2_O_3_ + 2 Li_2_CO_3_ + O_2_ = 4 LiMn_2_O_4_ + 2 CO_2_

On the basis of the above phase-evolution behaviour, the formation of dislocation structures in the LMO-C lattice can be further attributed to the mismatch in lithiation kinetics among different precursor phases. Such kinetic mismatch induces non-uniform crystal nucleation and growth, resulting in asynchronous local phase transformation and, ultimately, lattice mismatch and defect accumulation. To clarify this lithiation-kinetics mechanism, the relative phase fraction of LMO, denoted as *α*, was quantitatively determined during the reaction process through multiphase Rietveld refinement (Fig. 3b and d). The evolution of *α* as a function of reaction temperature *T* was then analysed to resolve the kinetic characteristics of different sintering stages (detailed analysis is provided in the SI).

After deconvolution of the d*α*/d*T* − *T* profiles, different phase-transformation pathways and reaction-control steps can be distinguished (Fig. 3e and f). Combined with Arrhenius analysis, the dominant kinetic control mechanism at each reaction stage can be further identified (Fig. 3g). Specifically, the formation of LMO-C proceeds through five major stages: (i) nucleation-controlled reaction between Mn_3_O_4_ and Li_2_CO_3_; (ii) chemically controlled reaction and solid-state diffusion between Mn_3_O_4_ and Li_2_CO_3_; (iii) chemically controlled oxidation of Mn_3_O_4_ to Mn_2_O_3_; (iv) nucleation-controlled reaction between Mn_2_O_3_ and Li_2_CO_3_; and (v) chemically controlled reaction and solid-state diffusion between Mn_2_O_3_ and Li_2_CO_3_. By contrast, the formation of LMO-H mainly follows a continuous lithiation pathway between Mn_3_O_4_ and Li_2_CO_3_, involving only the nucleation, growth and solid-state diffusion steps associated with the direct lithiation of Mn_3_O_4_, without an evident Mn_2_O_3_-mediated intermediate transformation.

The kinetic fitting further reveals markedly different reaction barriers for the two systems. The apparent activation energies (*E*_a_) for the individual stages involved in LMO-C formation are 187.58, 315.16, 238.99, 287.39 and 531.24 kJ mol^−1^, respectively (SI Table 4), all of which are substantially higher than those for LMO-H formation, namely 119.38, 158.34, 71.70 and 36.89 kJ mol^−1^. This difference indicates that Mn_3_O_4_-H provides more favourable lithiation kinetics and can be converted into spinel LMO with lower kinetic barriers. Notably, once the Mn_2_O_3_ intermediate is formed during the LMO-C reaction pathway, the activation energy for subsequent lithiation increases substantially. The emergence of Mn_2_O_3_ therefore slows the lithiation process and increases the difficulty of spinel-lattice reconstruction, leading to poorer crystal-structure quality in the final LMO product.

To exclude the influence of precursor particle size on the lithiation and sintering process, Mn_3_O_4_-C was further subjected to bead milling to reduce its particle size (Fig. S8 and S9). After particle-size reduction, the Li^+^ diffusion length in Mn_3_O_4_-C was effectively shortened, allowing lithiation to be completed before oxidation to Mn_2_O_3_ and thereby enabling the formation of LMO. Importantly, even when the precursor particle sizes are comparable, the lithiation of Mn_3_O_4_-C still occurs later than that of Mn_3_O_4_-H (Fig. S10). This finding suggests that, beyond the particle-size effect, Mn_3_O_4_-H possesses additional structural or interfacial features that further promote lithiation kinetics, as discussed in the following section.

### Electrochemical properties and ion transport kinetics

The electrochemical consequences of the different lithiation pathways were first evaluated using coin-type half cells assembled with LMO samples derived from different precursors. LMO-H exhibits excellent rate capability, delivering high reversible discharge capacities of 135.91, 131.65, 128.67, 126.49, 124.09 and 121.71 mAh g^−1^ at 1C, 2C, 5C, 10C, 20C and 30C, respectively (Fig. 4a). By contrast, LMO-H-800 shows inferior rate performance, further indicating that the LMO lattice obtained through the Mn_2_O_3_-related pathway possesses lower structural quality and is less favourable for rapid Li^+^ transport (Fig. 4b). LMO-C displays the poorest rate capability, which can be attributed to its higher density of lattice defects and larger particle size (Fig. 4c). After bead milling, the rate performance of LMO-C is partially improved, suggesting that particle-size reduction shortens the solid-state Li^+^ diffusion length and thereby enhances high-rate capability (Fig. 4d and e). Nevertheless, its rate performance remains inferior to that of LMO-H, further highlighting the critical role of early, continuous and uniform lithiation in enabling complete nucleation and growth of the spinel lattice.

**Fig. 4:**
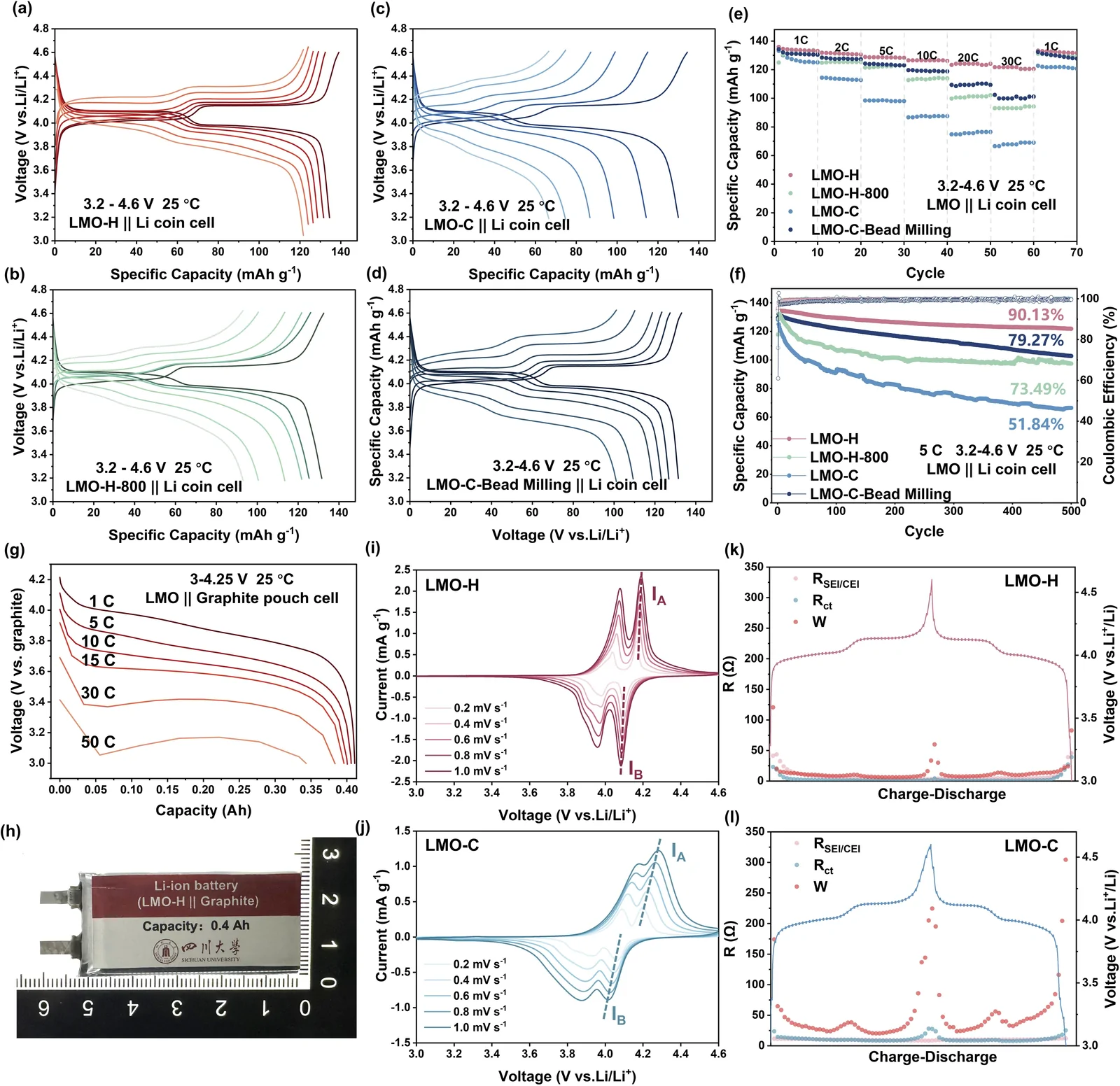
Comparative electrochemical performance and charge-storage kinetics of LMO synthesized from different precursors. (a–d) Rate-dependent charge–discharge voltage profiles of (a) LMO-H, (b) LMO-H-800, (c) LMO-C and (d) LMO-C-Bead Milling half cells. (e) Rate performance of LMO samples synthesized from different precursors. (f) Cycling performance of LMO samples synthesized from different precursors at 5C. (g) Discharge voltage profiles of the LMO-H‖graphite pouch cell at different current densities. (h) Optical photograph of the LMO-H‖graphite pouch cell. (i and j) Cyclic voltammograms of (i) LMO-H and (j) LMO-C at different scan rates. (k and l) Evolution of *R*_SEI_, *R*_ct_ and the *W* for (k) LMO-H and (l) LMO-C during electrochemical cycling.

The cycling stability of the four materials at a high current density of 5C further reveals pronounced differences in structural robustness. LMO-H retains 90.13% of its capacity after 500 cycles, substantially outperforming LMO-H-800 (73.49%), LMO-C-Bead Milling (79.27%) and LMO-C (51.84%) (Fig. 4f). This result indicates that LMO-H, formed through an early and continuous lithiation pathway, possesses a more stable crystal structure that can more effectively suppress Mn^3+^-related Jahn–Teller distortion and preserve structural integrity during prolonged cycling. To further assess its practical applicability, an LMO-H‖graphite pouch cell was assembled and tested at different current densities (Fig. 4g and h). The pouch cell delivers an initial capacity of 411 mAh and retains 83.53% of this capacity even under an ultrahigh discharge rate of 50C, confirming the fast charge–discharge capability of LMO-H in a practical cell configuration.

To clarify the kinetic origin of the superior electrochemical performance of LMO-H under ultrahigh current densities, the Li^+^ transport behaviours of LMO-H and LMO-C were analysed from cyclic voltammetry profiles collected at different scan rates. As shown in Fig. 4i and j, the main peak A corresponds to Mn oxidation, whereas peak B is associated with Mn reduction. LMO-H exhibits a larger fitted slope between peak current and the square root of scan rate, indicating improved apparent electrochemical kinetics (Fig. S11). Further galvanostatic intermittent titration technique (GITT) measurements show that LMO-H generally exhibits higher apparent Li^+^ diffusion coefficients (*D*_Li^+^_) than LMO-C during the charge–discharge process (Fig. S12), further supporting the enhanced Li^+^ diffusion kinetics of LMO-H. In addition, at a low scan rate of 0.1 mV s^−1^, LMO-H shows a smaller potential separation between the oxidation and reduction peaks, reflecting lower electrochemical polarization and higher reaction reversibility (Fig. S13a). Notably, the reduction peak at approximately 3.2 V is closely associated with oxygen vacancies in the spinel structure.^30,31^ The weaker intensity of this peak in LMO-H therefore further suggests a lower oxygen-vacancy concentration (Fig. S13).

The interfacial reaction and ion-transport resistances during charge–discharge cycling further reveal the kinetic differences between the LMO-H and LMO-C cells. The impedance responses obtained from *in situ* electrochemical impedance spectroscopy were further analysed using the distribution of relaxation times (DRT) method.^32–34^ The impedance-peak intensities of LMO-H cell remain substantially lower than those of LMO-C cell throughout the charge–discharge process (Fig. S14), indicating systematically reduced polarization and interfacial/transport resistance. Further integration of the corresponding relaxation-time regions reveals that the LMO-H cell consistently exhibits smaller resistance contributions associated with intermediate- and low-frequency electrochemical processes during cycling (Fig. 4k and l). These results indicate more favourable overall reaction and Li^+^ transport kinetics in the LMO-H‖Li system, which suppress electrochemical polarization and support its superior rate capability. By contrast, the larger impedance contributions observed for the LMO-C cell indicate more severe kinetic deterioration during cycling, thereby limiting rapid Li^+^ transport and contributing to its inferior rate performance.

### Structural stabilization mechanism

The fast and stable electrochemical response of LMO-H indicates that early continuous lithiation not only accelerates the formation of the spinel phase, but also establishes a spinel framework with a higher structural stability and more favourable Li^+^ migration pathways. To reveal how this precursor-regulated lithiation pathway stabilizes the final LMO lattice, the Mn valence state, oxygen-defect concentration, Li local chemical environment and Mn coordination structure were systematically analysed at both the precursor and product levels.

LMO-H contains a higher surface lattice-oxygen content and a larger fraction of Mn^4+^, indicating that more complete oxidation, nucleation and lattice reconstruction occur during sintering (Fig. 5a, b, d and e). Meanwhile, the paramagnetic signal associated with oxygen vacancies is markedly weaker in LMO-H than in LMO-C, further confirming its lower oxygen-defect concentration (Fig. 5c). The reduced concentration of oxygen vacancies helps preserve the integrity of the Mn–O framework, mitigate local structural distortion and provide a more continuous and stable structural framework for rapid Li^+^ migration. Moreover, in the solid-state ^7^Li NMR spectra, the main resonance at approximately 514 ppm in LMO-H splits and shifts towards a lower chemical shift relative to that of LMO-C, accompanied by the appearance of a shoulder peak at approximately 593 ppm (Fig. 5f). These features indicate a Li local environment enriched with neighbouring Mn^4+^.^35,36^ Such a local structure not only decreases the relative fraction of Jahn–Teller-active Mn^3+^ and thereby suppresses MnO_6_ octahedral distortion, but also creates a more favourable local environment for Li^+^ migration, enabling rapid Li^+^ transport under high current densities.^14,37^

**Fig. 5:**
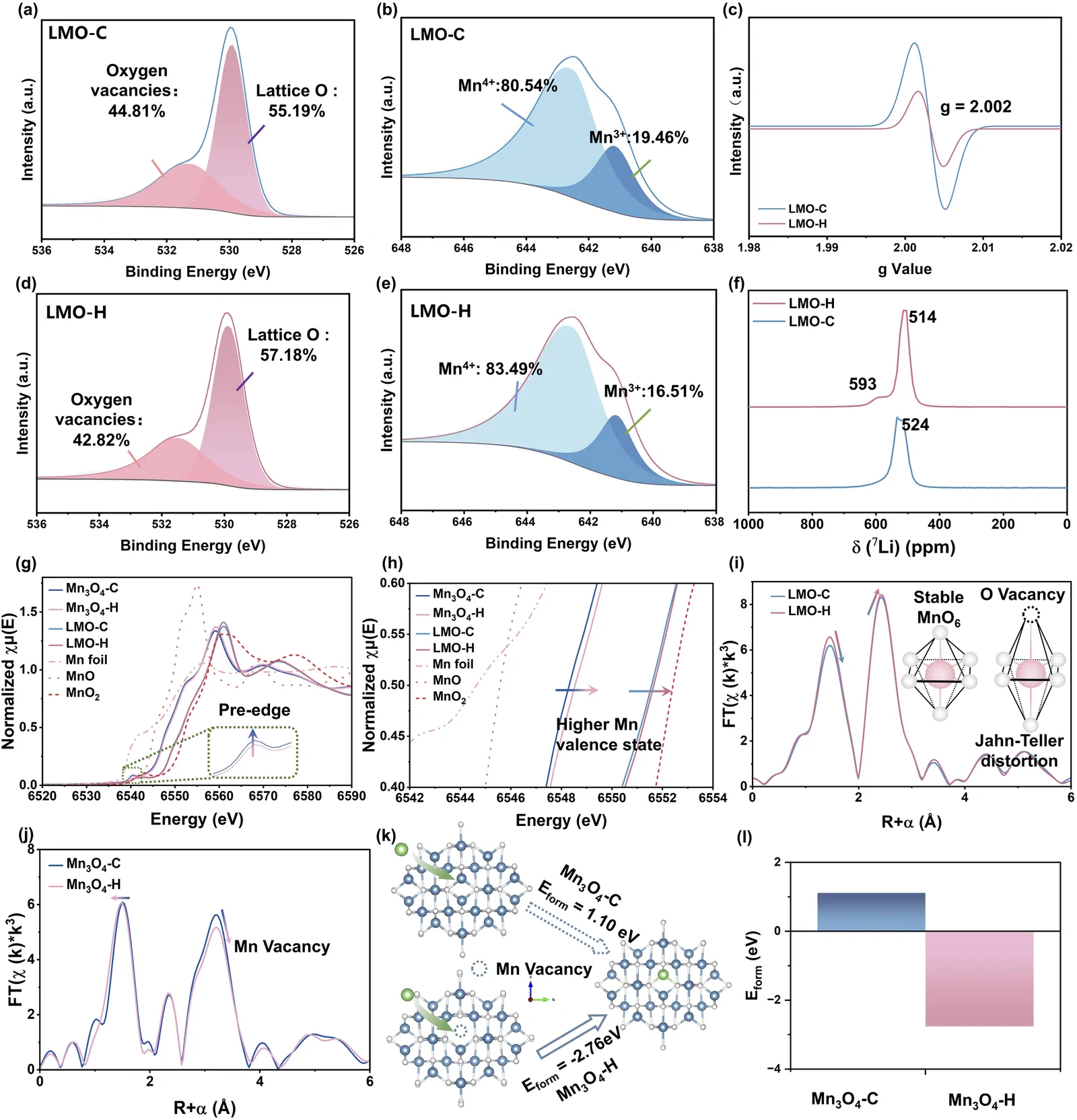
Mechanistic insight into the structural stability and precursor-dependent lithiation chemistry of LMO. (a and d) O 1s XPS spectra of (a) LMO-C and (d) LMO-H. (b and e) Mn 2p XPS spectra of (b) LMO-C and (e) LMO-H. (c) EPR spectra of LMO-H and LMO-C. (f) Solid-state ^7^Li NMR spectra of LMO-H and LMO-C. (g and h) Mn K-edge XAFS analysis of different samples: (g) normalized XAFS spectra and (h) enlarged XANES region. (i and j) Fourier-transformed EXAFS spectra in *R* space for (i) LMO-H and LMO-C and (j) Mn_3_O_4_-H and Mn_3_O_4_-C. (k) Schematic illustration of the lithiation pathways of Mn_3_O_4_-H and Mn_3_O_4_-C. (l) Comparison of the calculated formation energies along the lithiation pathways of Mn_3_O_4_-H and Mn_3_O_4_-C.

More detailed information on valence state and coordination structure further reveals the structural origin of the enhanced stability of LMO-H. For the Mn_3_O_4_ precursors, the Mn K-edge absorption edge of Mn_3_O_4_-H shifts to higher energy relative to that of Mn_3_O_4_-C, indicating a higher initial average Mn valence state (Fig. 5g and h). Meanwhile, the lower pre-edge intensity of Mn_3_O_4_-H suggests a more centrosymmetric Mn–O coordination environment with a lower degree of local distortion, reflecting a more stable Mn–O coordination structure.^38^ After lithiation, the absorption edge of LMO-H also shifts towards higher energy compared with that of LMO-C, further confirming the higher average Mn valence state in the final product. This result is consistent with the analyses of surface valence state, oxygen-defect concentration and Li local environment described above. The local coordination differences between the LMO products further reveal the origin of their distinct structural stability and Li^+^ transport capability. The *R*-space fitting results show that LMO-H has a shorter average Mn–O bond length and a higher Mn–O coordination number than LMO-C (Fig. 5i, S15–S18, SI Tables 5 and 6), indicating a more complete and stable MnO_6_ octahedral framework. This stable Mn–O coordination framework can effectively suppress local structural distortion during cycling and provides a structural basis for the excellent cycling stability of LMO-H. In addition, the longer Mn–Mn distance in LMO-H suggests a more open Li^+^ migration framework within the spinel lattice, which is favourable for rapid Li^+^ diffusion under high current densities (Fig. S19 and SI Table 7).

To further identify the intrinsic structural features of Mn_3_O_4_-H that promote the formation of high-quality LMO, the local coordination structures of the Mn_3_O_4_ precursors were also analysed. The Mn–O coordination shell of Mn_3_O_4_-H shifts towards a lower *R* value in the *R*-space profile compared with that of Mn_3_O_4_-C (Fig. 5j), indicating a more stable Mn–O coordination structure in Mn_3_O_4_-H. Notably, the Mn–Mn coordination shell of Mn_3_O_4_-H exhibits a lower peak intensity and shifts towards a higher *R* value, while the intensity corresponding to the Mn–Mn scattering path is weakened in the wavelet-transform map (Fig. S20). These features indicate a reduced Mn–Mn coordination number and an expanded local Mn–Mn coordination environment in Mn_3_O_4_-H, consistent with the presence of Mn deficiency. More direct structural information was obtained from XRD Rietveld refinement, which reveals partial Mn deficiencies at both crystallographically distinct Mn sites in Mn_3_O_4_-H. The vacancy fraction at the tetrahedral 4a site is approximately 1.9%, whereas that at the octahedral 8d site is only approximately 0.5% (Fig. S21 and SI Tables 8–10), indicating a preferential deficiency of tetrahedral Mn^2+^. To further clarify this site preference, the formation energies of Mn vacancies at the two sites were calculated under identical computational conditions and chemical-potential references. The formation energies of vacancies at the tetrahedral Mn^2+^ and octahedral Mn^3+^ sites are 3.872 and 4.506 eV respectively, demonstrating that Mn-vacancy formation is thermodynamically more favorable at the tetrahedral 4a site. Moreover, such cation deficiency is expected to be accompanied by partial oxidation of Mn for charge compensation (Fig. S22 and SI Table 11), consistent with the slightly higher average Mn valence of Mn_3_O_4_-H revealed by XANES analysis. This defect-regulated precursor structure can provide more accessible solid-state diffusion pathways for Li^+^ during sintering, thereby accelerating lithiation nucleation and lattice reconstruction.

The calculated formation energies further support this mechanism. The Mn-vacancy-containing Mn_3_O_4_-H structure exhibits a much lower lithiation formation energy of −2.76 eV, compared with 1.10 eV for Mn_3_O_4_-C, indicating that the presence of Mn vacancies substantially improves the thermodynamic feasibility of lithiation (Fig. 5k and l). As a result, Mn_3_O_4_-H can undergo rapid lithiation nucleation at a lower reaction temperature and form LMO through a continuous lithiation pathway, yielding a product with higher lattice integrity, a lower oxygen-defect concentration and a more stable Mn–O framework. This structural-evolution mechanism, from a defect-regulated precursor structure to a stabilized product lattice, provides the fundamental origin of the superior rate capability and cycling stability of LMO-H.

### Investigation of material failure mechanisms

Finally, the failure mechanisms of LMO with different structural characteristics were further investigated by analysing the impedance evolution and structural degradation after cycling. During prolonged cycling, LMO-C cell undergoes a pronounced increase in overall electrochemical impedance, indicating progressively hindered interfacial charge transfer and bulk ion transport (Fig. 6b). Further DRT analysis reveals that multiple relaxation processes of the LMO-C cell increase continuously during cycling (Fig. 6c and S23), which can be mainly attributed to the higher density of lattice defects, dislocations and local structural distortions in the pristine material. These structural imperfections amplify local stress during repeated Li^+^ insertion and extraction, impede charge transfer and Li^+^ migration, and ultimately lead to rapid capacity decay and poor cycling stability. By contrast, all impedance components of LMO-H cell remain low and relatively stable over 500 cycles, indicating that its spinel lattice and interfacial structure are better preserved during long-term cycling (Fig. 6a). This stable impedance response is closely associated with its lower oxygen-defect concentration, more intact Mn–O framework and more continuous Li^+^ transport network, further confirming that the early continuous lithiation pathway enhances the structural stability of LMO during cycling.

**Fig. 6:**
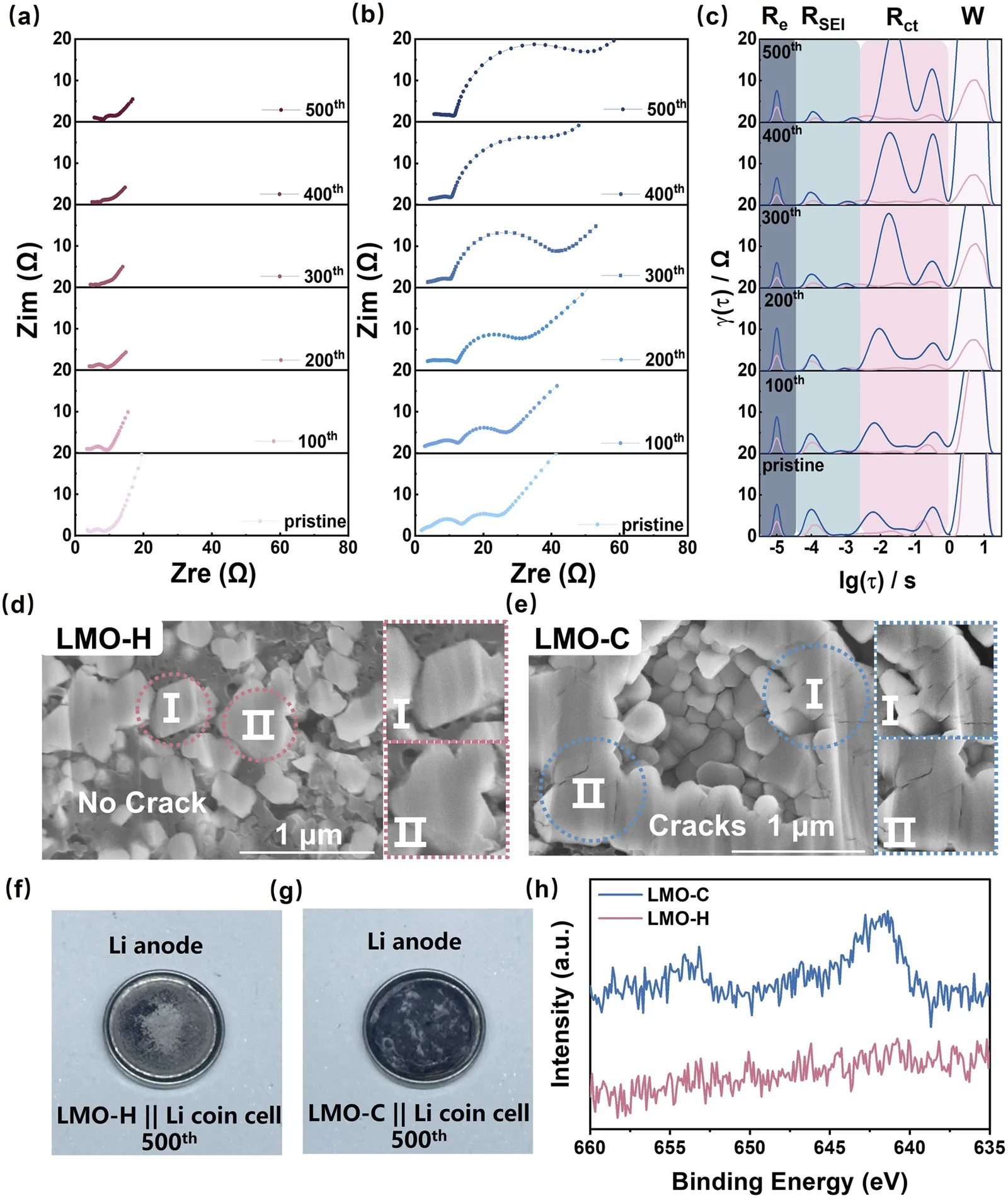
Degradation-mechanism characterization of LMO electrodes during electrochemical cycling. (a and b) Electrochemical impedance spectra of (a) LMO-H and (b) LMO-C after selected numbers of cycles. (c) DRT profiles of LMO-H and LMO-C after different numbers of cycles. (d and e) Cross-sectional SEM images of (d) LMO-H and (e) LMO-C electrodes after 500 cycles. (f and g) Lithium anodes recovered from the disassembled coin cells after 500 cycles for (f) LMO-H and (g) LMO-C. (h) XPS signals of Mn on the lithium anodes corresponding to LMO-C and LMO-H cells.

The post-cycling structural evolution further reveals the origin of the rapid failure of LMO-C. After prolonged cycling, evident internal cracks are observed within LMO-C particles (Fig. 6d and e), indicating severe stress accumulation and structural damage during electrochemical cycling. Meanwhile, the Li anode recovered from the LMO-C half-cell exhibited a distinctly darkened surface, consistent with more extensive interfacial side reactions (Fig. 6f and g). In addition, the corresponding Li anode showed a markedly stronger Mn-related XPS signal than that recovered from the LMO-H half-cell (Fig. 6h), consistently, ICP-OES analysis revealed a substantially higher amount of Mn deposited on the Li anode from the LMO-C half-cell (Fig. S24), collectively indicating more severe Mn dissolution, migration, and subsequent deposition during prolonged cycling. Specifically, the combined effects of stronger Jahn–Teller distortion, Mn dissolution, oxygen vacancy migration and dislocation defects induce higher stress concentration around locally mismatched lattice regions, thereby promoting crack initiation and propagation. The formation of cracks, together with continuous Mn dissolution, not only disrupts continuous electronic and ionic transport pathways, but also accelerates electrolyte penetration and interfacial side reactions, further increasing electrochemical polarization and accelerating capacity fading. In contrast, the more complete and stable lattice structure of LMO-H effectively mitigates stress accumulation during cycling, suppresses crack formation and Mn dissolution, and maintains fast and stable charge-transfer and Li^+^ migration kinetics, resulting in markedly improved cycling stability.

## Conclusions

In summary, this work reveals the decisive role of lithiation pathways in governing lattice formation, defect evolution and electrochemical performance during the solid-state synthesis of LMO. For the commercially relevant Mn_3_O_4_ precursor, the conversion to spinel LMO is dictated by the competition between lithiation and oxidation. The Mn_3_O_4_ precursor containing intrinsic Mn defects exhibits a lower lithiation formation energy, enabling rapid and homogeneous early-stage lithiation while suppressing the formation of the Mn_2_O_3_ intermediate. As a result, LMO with higher lattice integrity, a lower oxygen-vacancy concentration and a more robust Mn–O framework is obtained, leading to superior rate capability and cycling stability. By contrast, delayed lithiation introduces the Mn_2_O_3_ intermediate and triggers competitive lithiation among different phases. The mismatch in lithiation thermodynamics and kinetics results in asynchronous crystal nucleation and growth, inducing dislocations, local lattice distortion and stress accumulation that ultimately cause particle cracking, hinder Li^+^ transport and accelerate capacity degradation during cycling. Rather than relying on conventional strategies based on elemental doping or surface modification, this work establishes a precursor-regulated synthesis strategy through lithiation-pathway engineering, providing new mechanistic insights and general design principles for the controllable synthesis of high-performance LMO and other manganese-based cathode materials.

## Author contributions

Rui-Xiang Wang: conceptualization, writing – original draft, writing – review & editing, formal analysis, visualization, validation, data curation, investigation; Yu-Qi Zhou: software, formal analysis; Jia-Qi Huang: software; Ce-Heng Duan: software; Jia-Jie Zhang: formal analysis; Le-Yi Zhong: visualization; Zhi-Zhong Shen: investigation; Xue-Chun Huang: investigation; Bin He: visualization; Xiao-Juan Chen: supervision, funding acquisition; Tian-Yuan Xiao: resources, funding acquisition, methodology, conceptualization; Dan Xiao: resources, supervision, funding acquisition, project administration, conceptualization; Yan Meng: funding acquisition, project administration, data curation, writing – review & editing, conceptualization, methodology, supervision.

## Conflicts of interest

There are no conflicts to declare.

## Supplementary Material

SC-OLF-D6SC06403E-s001

## Data Availability

The data supporting this article have been included as part of the supplementary information (SI). Supplementary information: additional experimental details, materials, methods, and characterization, including XRD patterns and Rietveld refinement results, SEM and HR-TEM images of the samples and calcination intermediates, ICP-OES compositional analysis, GITT measurements, CV and EIS-DRT data, curve fitting of Mn K-edge EXAFS data, wavelet transform images, computational methods and results, including defect energetics, Bader charge analysis of Mn_3_O_4_ defect structures, and Li^+^ migration simulations, as well as detailed specifications for pouch-cell. See DOI: https://doi.org/10.1039/d6sc06403e.
