# Supplementary material for: Mn vacancies in Mn3O4: steering lithiation kinetics during solid-state synthesis toward ultrastable spinel cathode materials
Source: Chem Sci. 2026 Sep 23;17(43):21019–30. doi: 10.1039/d6sc06403e (PMC13617447; doi:10.1039/d6sc06403e)
Supplement: Supplementary file 1 [file SC-017-D6SC06403E-s001.pdf]

Supplementary Information (SI) for Chemical Science.
This journal is © The Royal Society of Chemistry 2026

# Supplementary Information

# Mn vacancies in $Mn_3O_4$: Steering lithiation kinetics during solid-state synthesis toward ultrastable spinel cathode materials.

Rui-Xiang Wang, Yu-Qi Zhou, Jia-Qi Huang, Ce-Heng Duan, Jia-Jie Zhang, Le-Yi Zhong, Zhi-Zhong Shen, Xue-Chun Huang, Bin He, Xiao-Juan Chen*, Tian-Yuan Xiao*, Dan Xiao, Yan Meng*

School of Chemical Engineering, Institute of New Energy and Low-Carbon Technology, Sichuan University, Chengdu 610065, Sichuan, China

Email: mengyan@scu.edu.cn

# Table of Contents

## Supplementary Tables

Supplementary Table 1. Mn contents of $Mn_3O_4$-H and $Mn_3O_4$-C determined by ICP-OES ........................... 4

Supplementary Table 2. Li/Mn molar ratios of the synthesized LMO samples determined by ICP-OES ....... 4

Supplementary Table 3. Lattice parameters of LMO-H and LMO-C. ............................................................. 5

Supplementary Table 4. Kinetic parameters for each lithiation reaction step ............................................. 9

Supplementary Table 5. Curve-fit Parameters for Mn K-edge EXAFS for LMO-H ..................................... 22

Supplementary Table 6. Curve-fit Parameters for Mn K-edge EXAFS for LMO-C ..................................... 24

Supplementary Table 7. Relationship between unit cell parameters and Mn–Mn bond lengths .................. 26

Supplementary Table 8. Lattice parameters of $Mn_3O_4$-C and $Mn_3O_4$-H ........................................................ 28

Supplementary Table 9. Structure parameter obtained from Rietveld refinement of $Mn_3O_4$-C ................... 28

Supplementary Table 10. Structure parameter obtained from Rietveld refinement of $Mn_3O_4$-H ................. 28

Supplementary Table 11. Average Bader charges of various types of atoms ................................................ 29

Supplementary Table 12. Detailed specifications for pouch-cell ................................................................. 30

**Supplementary Figures** ....................................................................................................................

Figure S1. XRD patterns of different precursors ........................................................................................ 6

Figure S2. SEM images of different temperature stages during the sintering process ................................... 7

Figure S3. HR-TEM images of intermediates during the sintering process of LMO-C. ................................ 10

Figure S4. HR-TEM images of intermediates during the sintering process of LMO-C ................................ 11

Figure S5. HR-TEM and FFT images of LMO-C .......................................................................................... 12

Figure S6. HR-TEM and FFT images of LMO-H .......................................................................................... 13

Figure S7. HR-TEM and FFT images of LMO-H-800 .................................................................................. 14

Figure S8. SEM images of different precursors. .......................................................................................... 15

Figure S9. SEM images of different products ............................................................................................... 16

Figure S10. In-situ high-temperature XRD contour maps of the $Mn_3O_4$-C-Bead Milling/$Li_2CO_3$ mixtures . 17

Figure S11. Fitted Curve of Peak Current in Cyclic Voltammetry ............................................................... 18

Figure S12. $Li^+$ diffusion coefficients of LMO-C and LMO-H battery systems measured via GITT ............ 19

Figure S13. CV curves of different products at a scan rate of 0.1 mV $s^{-1}$ .................................................... 20

Figure S14. In-situ EIS-DRT images ............................................................................................................ 21

Figure S15. Mn K-edge EXAFS and the curve-fit for LMO-H, shown in k-space ....................................... 22

Figure S16. Mn K-edge EXAFS and the curve-fit for LMO-H, shown in R-space ...................................... 23

Figure S17. Mn K-edge EXAFS and the curve-fit for LMO-C, shown in k-space ........................................24

Figure S18. Mn K-edge EXAFS and the curve-fit for LMO-C, shown in R-space........................................25

Figure S19. Mean squared displacement (MSD) of $Li^+$ as a function of LMO unit cell parameters .............26

Figure S20. EXAFS wavelet transform images of the different precursors ..................................................27

Figure S21. XRD refinement patterns of different precursors.......................................................................28

Figure S22. Variation of Bader charges for individual atoms with distance from the defect site under different Mn defect structures ..........................................................................................................29

Figure S23. DRT analyses of the LMO||Li cell before and after replacement of the cycled Li-metal counter electrode with fresh Li. ..........................................................................................................32

Figure S24. Mn content in lithium anode measured by ICP-OES .................................................................33

**Solid-state reaction kinetic analysis** ............................................................................................8

**Li-electrode replacement experiment and DRT analysis.** ..............................................................31

**Cell assembly and electrochemical Testing** ...................................................................................34

**Materials characterizations**............................................................................................................37

**First-principles calculation method**...............................................................................................39

**Reference**........................................................................................................................................41

Supplementary Table 1. Mn contents of $Mn_3O_4$-H and $Mn_3O_4$-C determined by ICP-OES.

| Name | Sample | Weight (mg) | Mn (mg/L) | Mn content |
|---|---|---|---|---|
| **$Mn_3O_4$-H** | 1 | 20.2 | 5.75 | 71.16% |
| | 2 | 20.1 | 5.73 | 71.27% |
| | 3 | 20.2 | 5.76 | 71.29% |
| | Average | - | - | 71.24% |
| **$Mn_3O_4$-C** | 1 | 20.3 | 5.81 | 71.55% |
| | 2 | 20.2 | 5.79 | 71.66% |
| | 3 | 20.1 | 5.75 | 71.52% |
| | Average | - | - | 71.58% |

Supplementary Table 2. Li and Mn contents and Li/Mn molar ratios of the synthesized LMO samples determined by ICP-OES.

| Name | Sample | Li (mg/L) | Mn (mg/L) | Li/Mn |
|---|---|---|---|---|
| **LMO-H** | 1 | 2.12 | 33.4 | 0.501761 |
| | 2 | 2.06 | 32.5 | 0.502461 |
| | 3 | 2.09 | 32.9 | 0.502879 |
| | Average | - | - | 0.502367 |
| **LMO-C** | 1 | 2.15 | 33.9 | 0.502056 |
| | 2 | 2.27 | 35.8 | 0.501945 |
| | 3 | 2.05 | 32.3 | 0.502417 |
| | Average | - | - | 0.502139 |
| **LMO-H-800** | 1 | 2.11 | 33.2 | 0.503104 |
| | 2 | 2.18 | 34.3 | 0.503125 |
| | 3 | 1.94 | 30.5 | 0.503518 |
| | Average | - | - | 0.503249 |
| **LMO-C-Bead Milling** | 1 | 2.09 | 33.0 | 0.501355 |
| | 2 | 2.28 | 35.9 | 0.502752 |
| | 3 | 2.17 | 34.2 | 0.502281 |
| | Average | - | - | 0.502129 |

Supplementary Table 3. Lattice parameters of LMO-H and LMO-C.

| Samples | a/b/c (Å) | V (Å$^3$) |
|---|---|---|
| LMO-H | 8.24295 | 560.077333 |
| LMO-C | 8.23914 | 559.302000 |

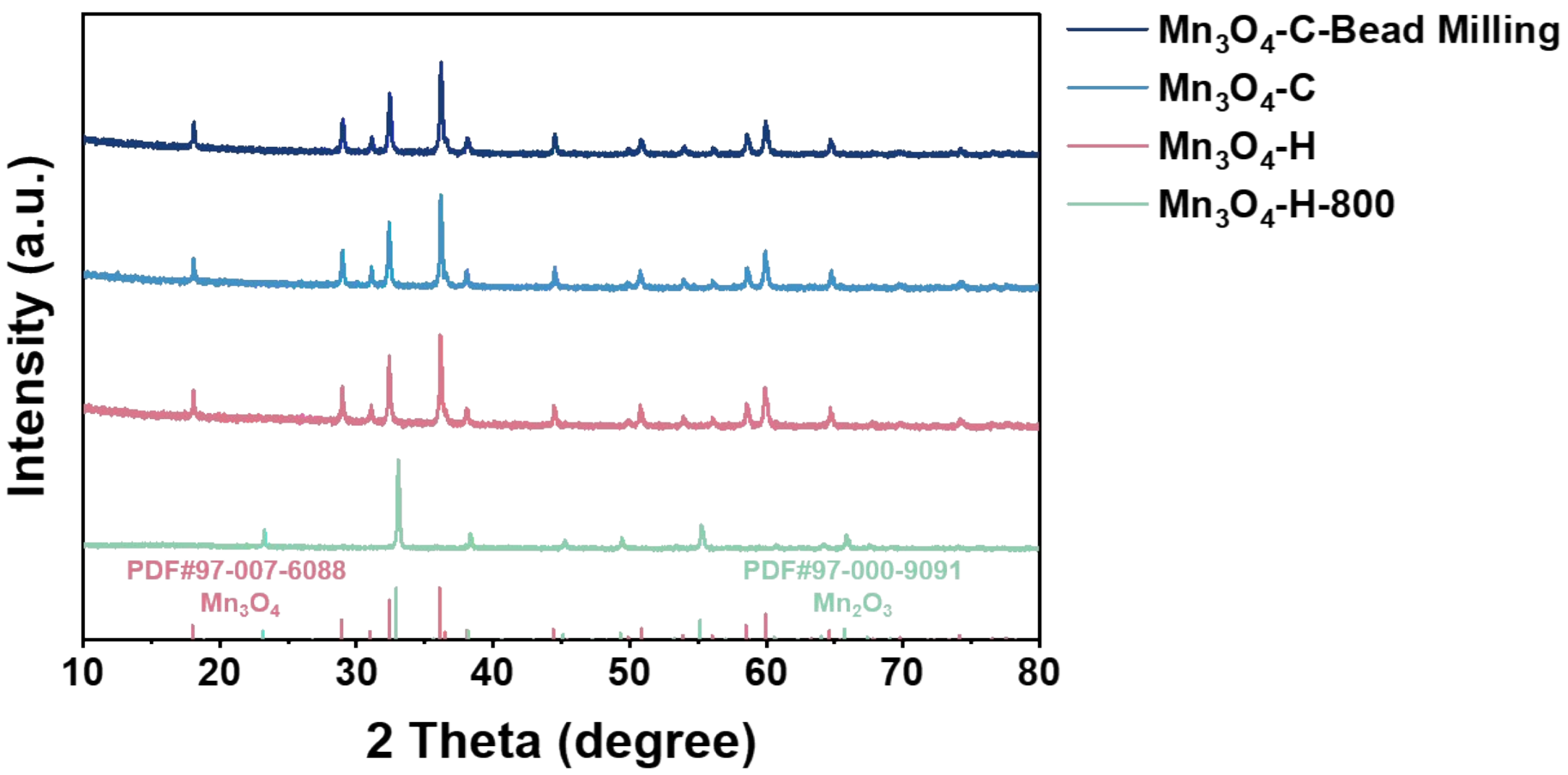


Figure S1. XRD patterns of $Mn_3O_4$-H, $Mn_3O_4$-H-800, $Mn_3O_4$-C, $Mn_3O_4$-C-Bead Milling.

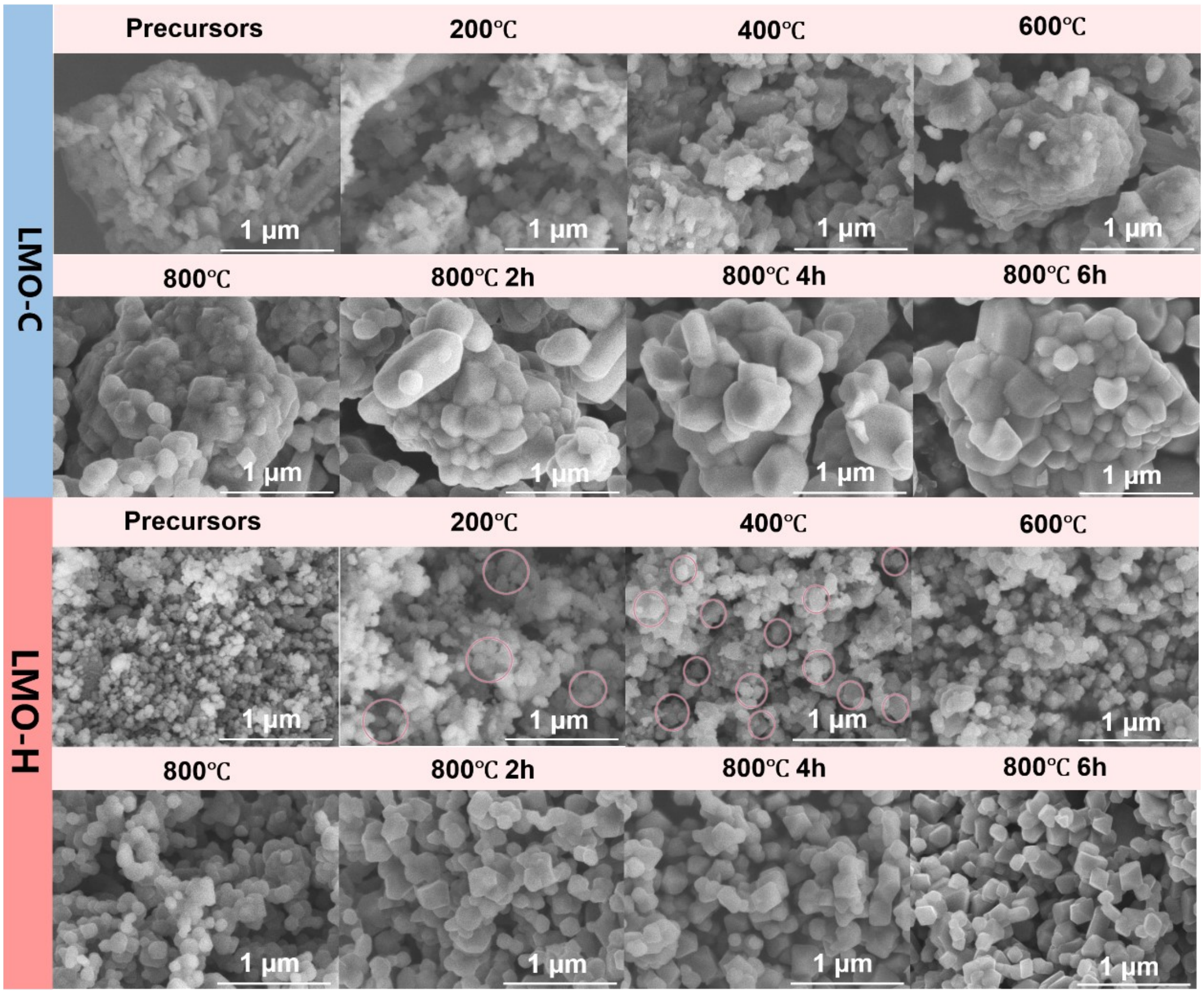


Figure S2. SEM images of LMO-C and LMO-H at different temperature stages during the sintering process.

**Solid-state reaction kinetic analysis:**[1]

According to the general rate law for solid-state reactions, the conversion kinetics under isothermal conditions can be expressed as follows:

$$\frac{d\alpha}{dt} = Aexp\left( - \frac{E_a}{RT}\right)f(\alpha)$$

where $\alpha$ denotes the conversion degree at a given time t, A is the pre-exponential factor, $E_a$ is the apparent activation energy, R is the gas constant, T is the absolute temperature and $f(\alpha)$ represents the reaction model.

For a non-isothermal process, the heating rate β is introduced, giving:

$$\frac{d\alpha}{dT} = \frac{A}{\beta}exp\left( - \frac{E_a}{RT}\right)f(\alpha)$$

Accordingly, this equation can be rearranged as:

$$ln\left(\frac{d\alpha}{dT}\right) - lnf(a) = ln\left(\frac{A}{\beta}\right) - \frac{E_a}{RT}$$

The kinetic model $f(\alpha)$ can be described using the following general form: [2]

$$f(a) = c(1 - a)^n a^m$$

where c is a constant, and m, n are empirical reaction-order parameters associated with the conversion process.

Substitution of this expression into the non-isothermal kinetic equation yields:

$$ln\left(\frac{d\alpha}{dT}\right) - ln((1 - a)^n a^m) = ln\left(\frac{cA}{\beta}\right) - \frac{E_a}{RT}$$

This expression was used as the fundamental equation to extract kinetic information from the experimentally determined $\alpha$-T data at a fixed heating rate β. The values of m and n were selected by optimizing the linearity of the corresponding Arrhenius-type plots, which enabled identification of the dominant kinetic characteristics governing the reaction process. Once m and n were determined, the apparent activation energy $E_a$ was obtained from the slope of the linear fit.

Supplementary Table 4. Kinetic parameters for each lithiation reaction step. D3 represents the 3D diffusion-Jander model: $[3(1-\alpha)^{2/3}]/[2(1-(1-\alpha)^{1/3})]$, F3 represents the third-reaction-order model: $(1-\alpha)^3$.[3]

<table>
<tr><th>Samples</th><th>Step</th><th>Reaction Model $f(\alpha)$</th><th>Activation Energy ($E_a$) ($kJ\cdot mol^{-1}$)</th></tr>
<tr><td rowspan="4">LMO-H</td><td>1</td><td>$[3(1-\alpha)^{2/3}]/[2(1-(1-\alpha)^{1/3})]$</td><td>119.38</td></tr>
<tr><td>2</td><td rowspan="3">$(1-\alpha)^3$</td><td>158.34</td></tr>
<tr><td>3</td><td>71.70</td></tr>
<tr><td>4</td><td>36.89</td></tr>
<tr><td rowspan="5">LMO-C</td><td>1</td><td>$[3(1-\alpha)^{2/3}]/[2(1-(1-\alpha)^{1/3})]$</td><td>187.58</td></tr>
<tr><td>2</td><td rowspan="2">$(1-\alpha)^3$</td><td>315.16</td></tr>
<tr><td>3</td><td>238.99</td></tr>
<tr><td>4</td><td>$[3(1-\alpha)^{2/3}]/[2(1-(1-\alpha)^{1/3})]$</td><td>287.39</td></tr>
<tr><td>5</td><td>$(1-\alpha)^3$</td><td>531.24</td></tr>
</table>

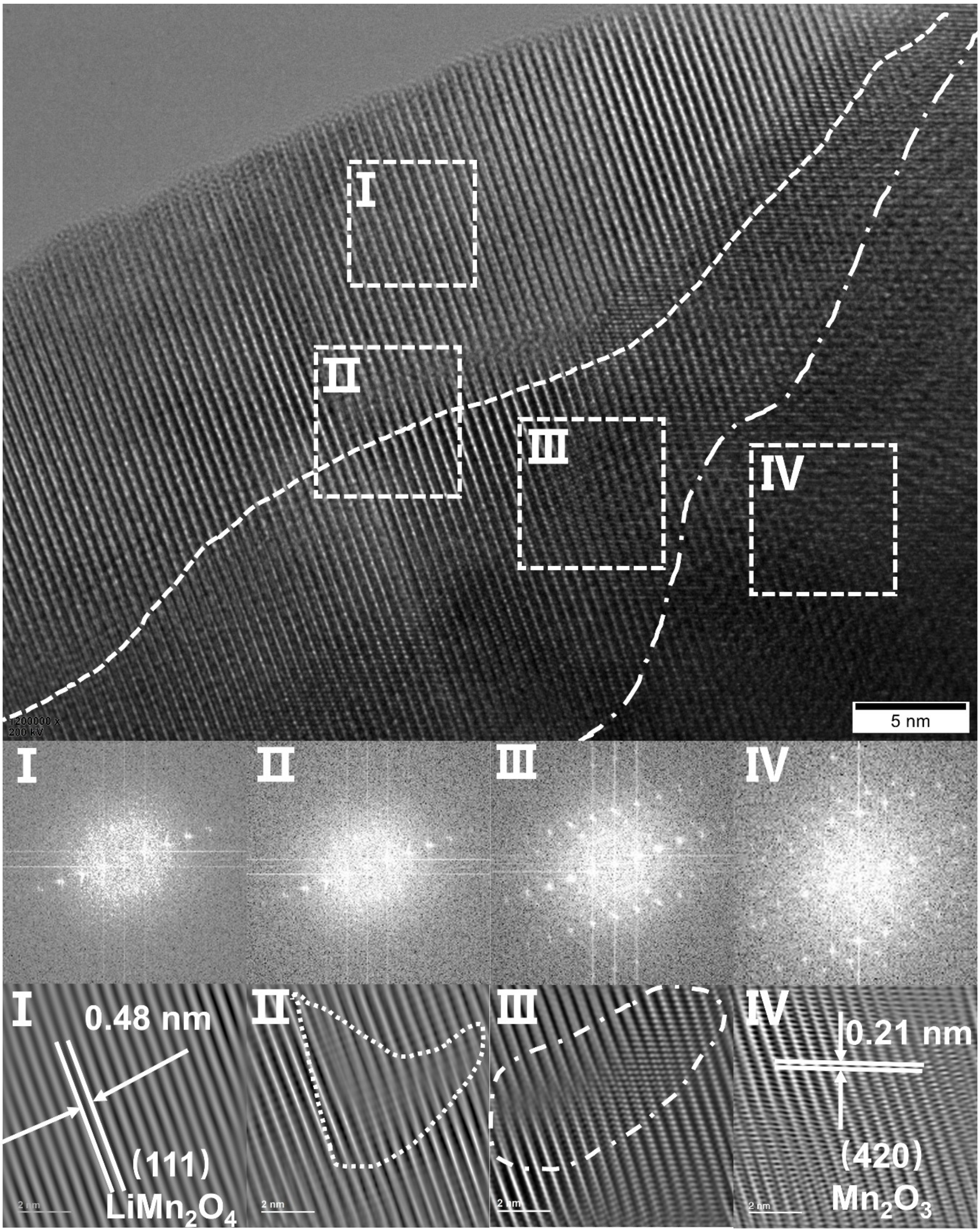


Figure S3. HR-TEM images of intermediates during the sintering process of LMO-C, along with FFT and IFFT images of selected regions.

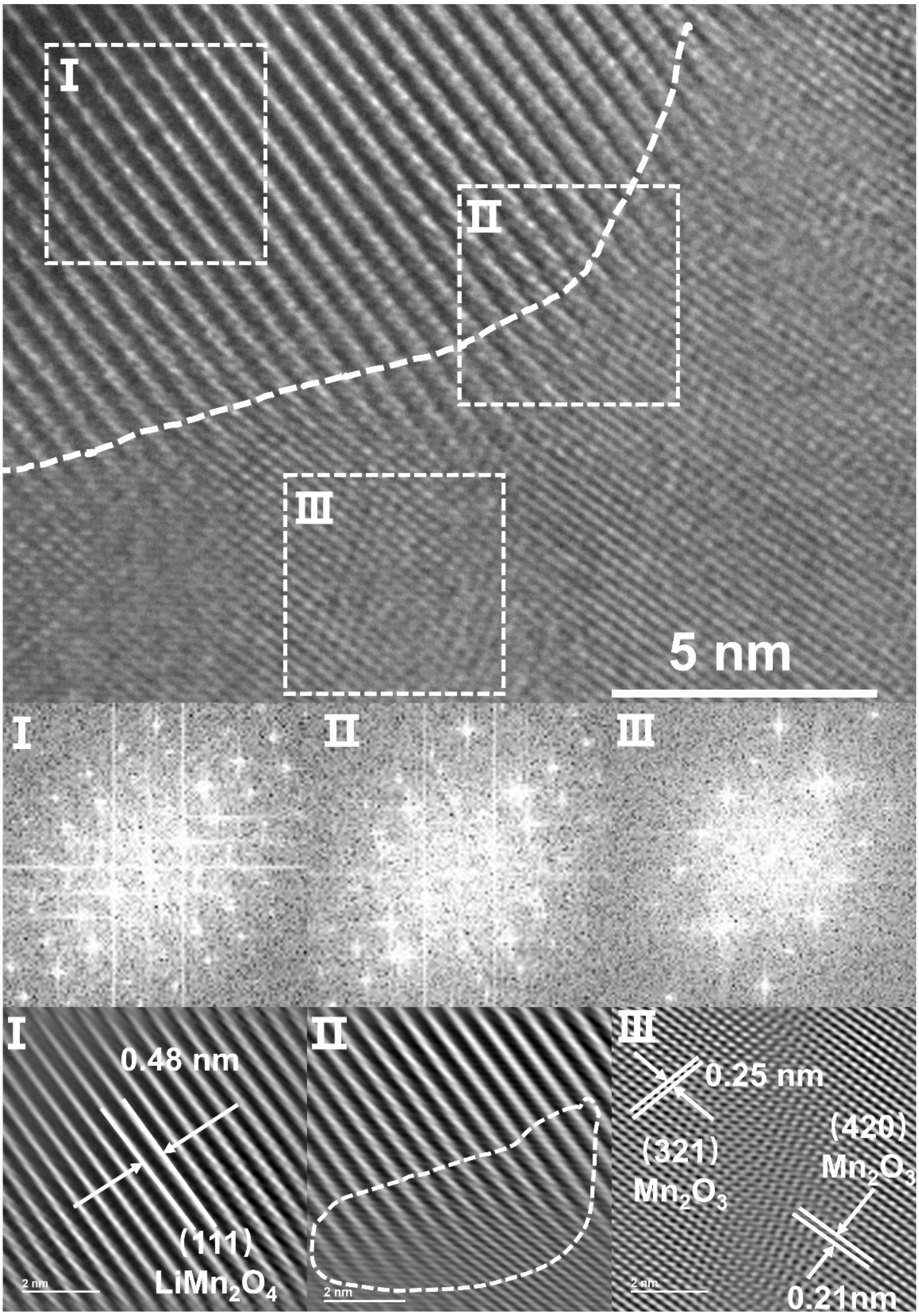


Figure S4. HR-TEM images of intermediates during the sintering process of LMO-C, along with FFT and IFFT images of selected regions.

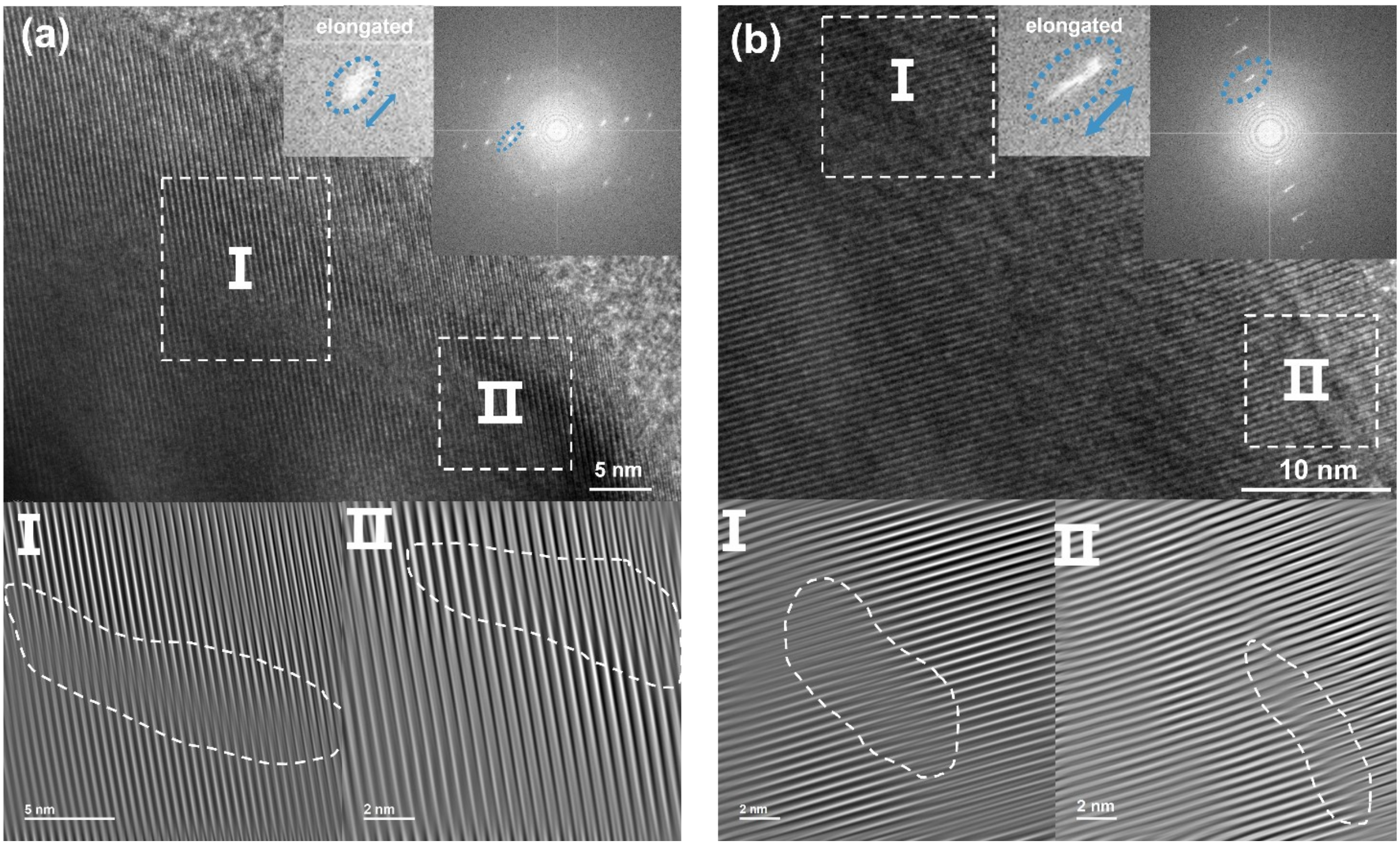


Figure S5. HR-TEM and FFT images of LMO-C, along with IFFT images of selected regions. (a) Near-surface region of the particle; (b) core region of the particle.

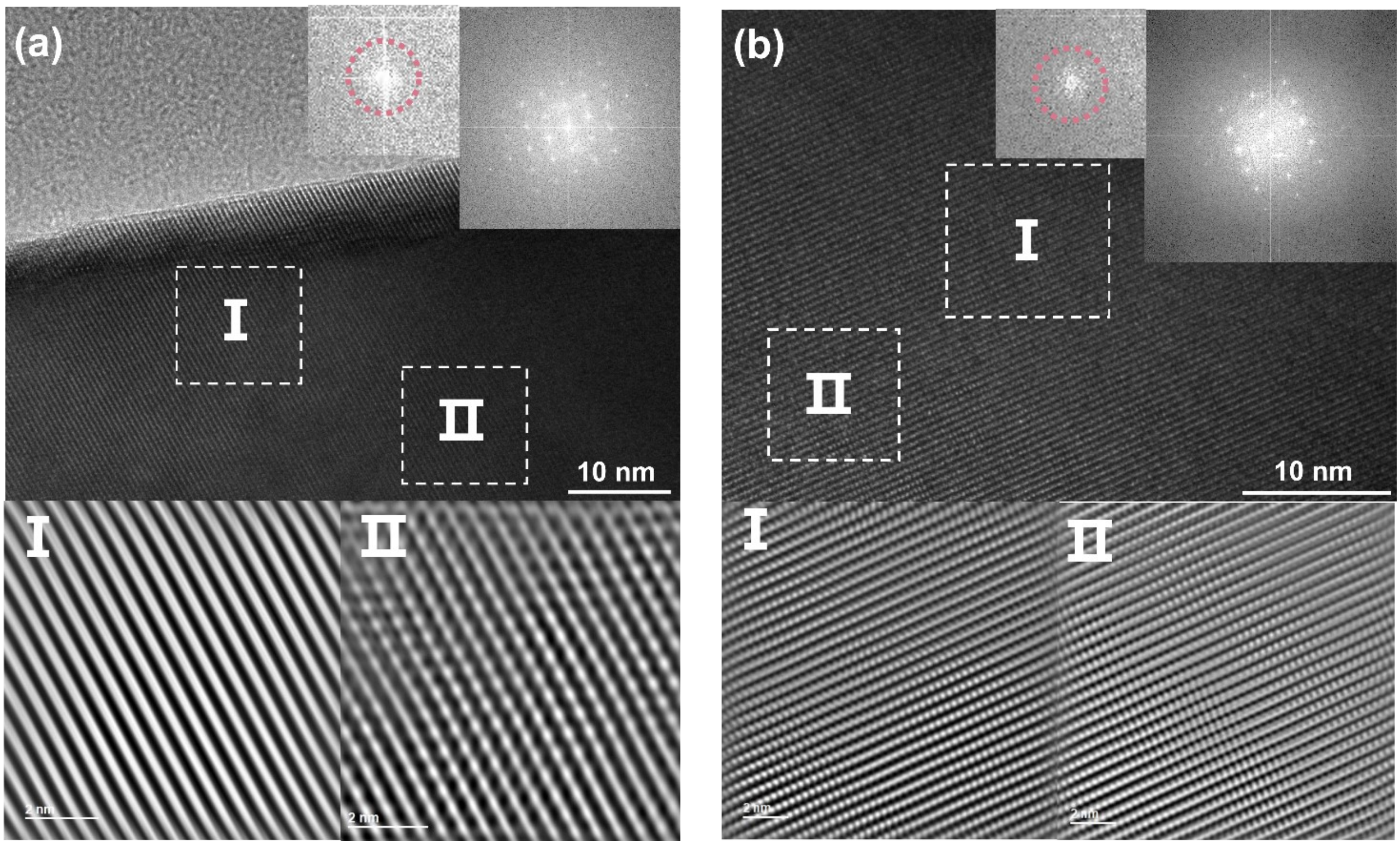


Figure S6. HR-TEM and FFT images of LMO-H, along with IFFT images of selected regions. (a) Near-surface region of the particle; (b) core region of the particle.

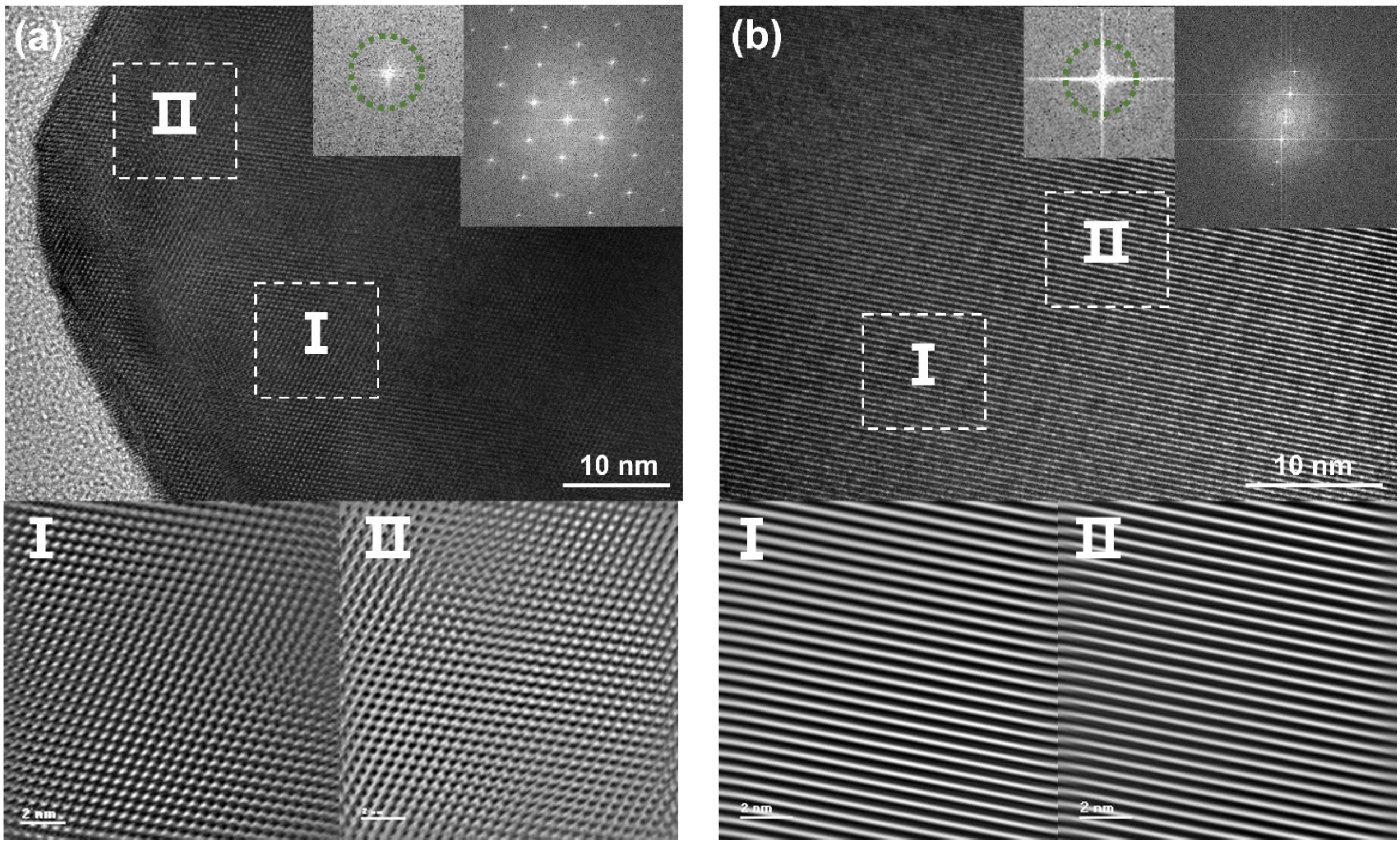


Figure S7. HR-TEM and FFT images of LMO-H-800, along with IFFT images of selected regions.

(a) Near-surface region of the particle; (b) core region of the particle.

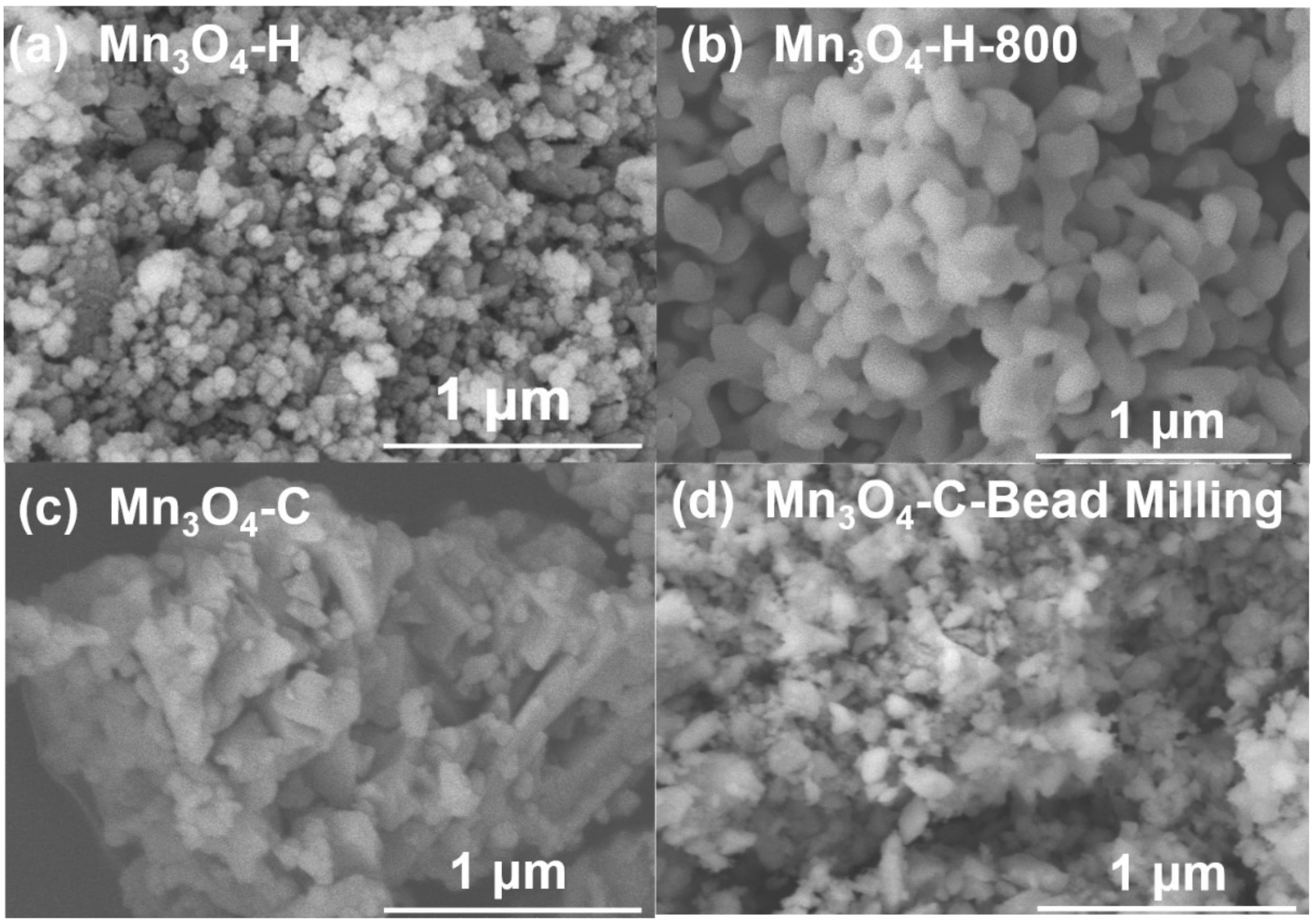


Figure S8. SEM images of different precursors. (a)$Mn_3O_4$-H, (b) $Mn_3O_4$-H -800, (c) $Mn_3O_4$-C, (d) $Mn_3O_4$-C-Bead Milling.

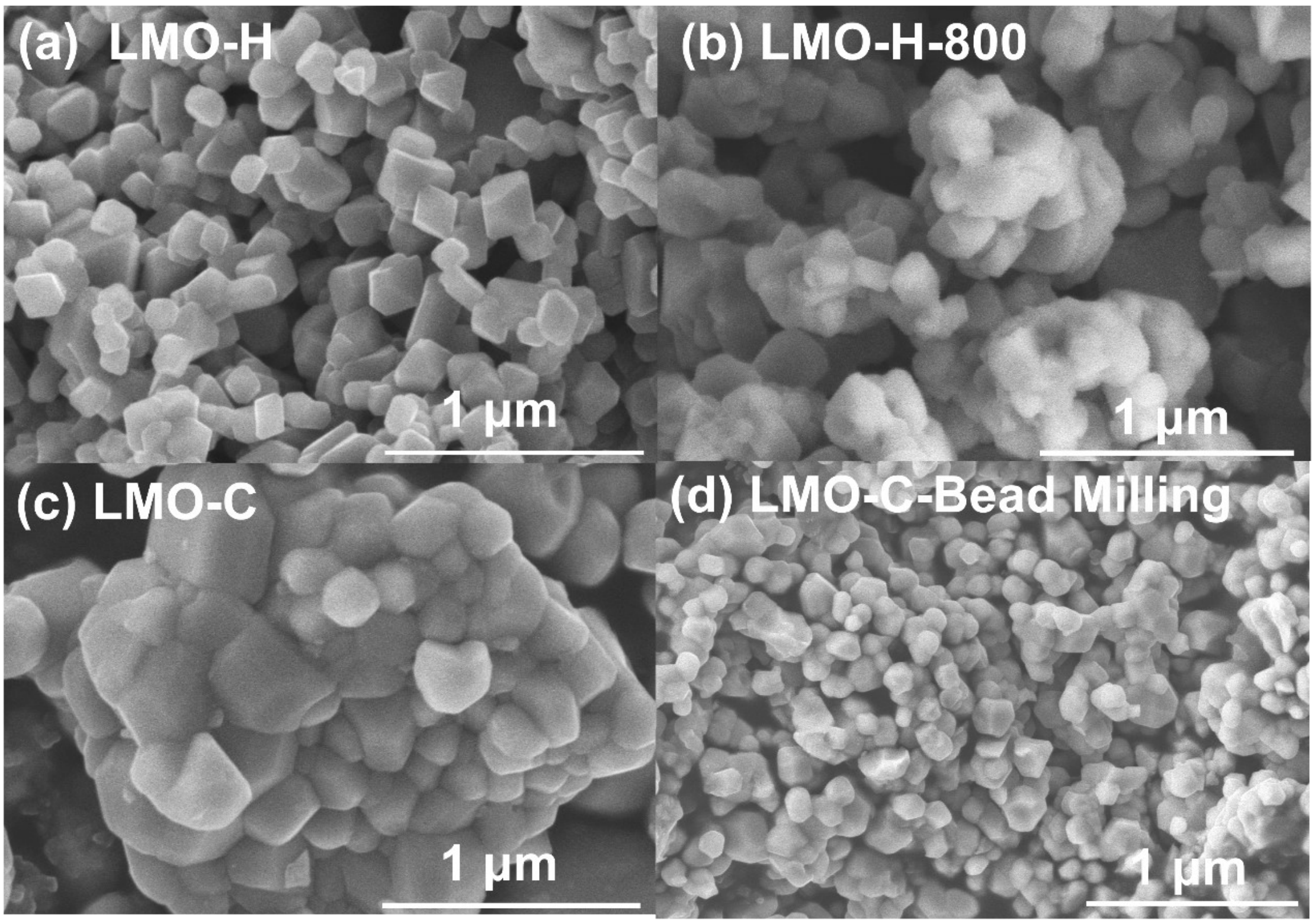


Figure S9. SEM images of LMOs synthesized from different precursors. (a) LMO-H, (b) LMO-H-800, (c) LMO-C, (d) LMO-C-Bead Milling.

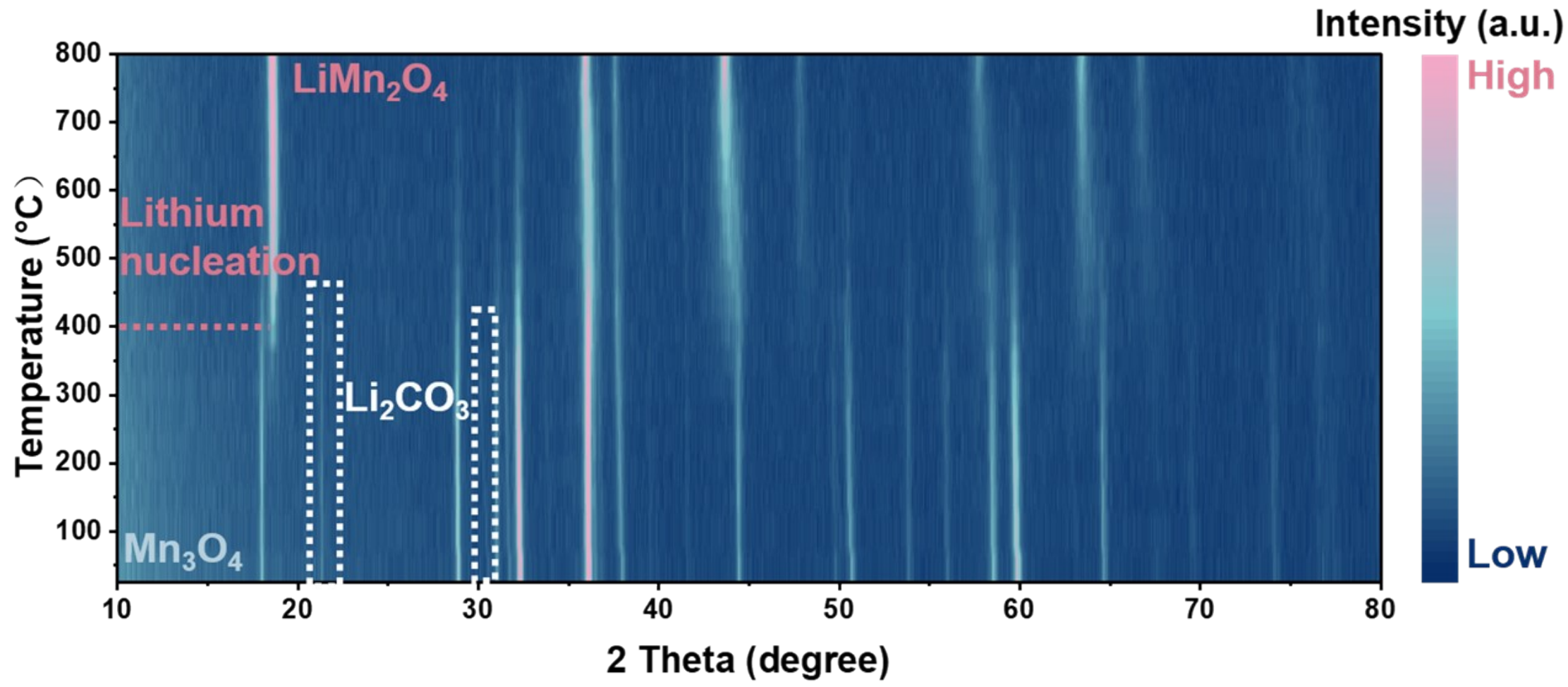


Figure S10. In-situ high-temperature XRD contour maps of the $Mn_3O_4$-C-Bead Milling/$Li_2CO_3$ mixtures.

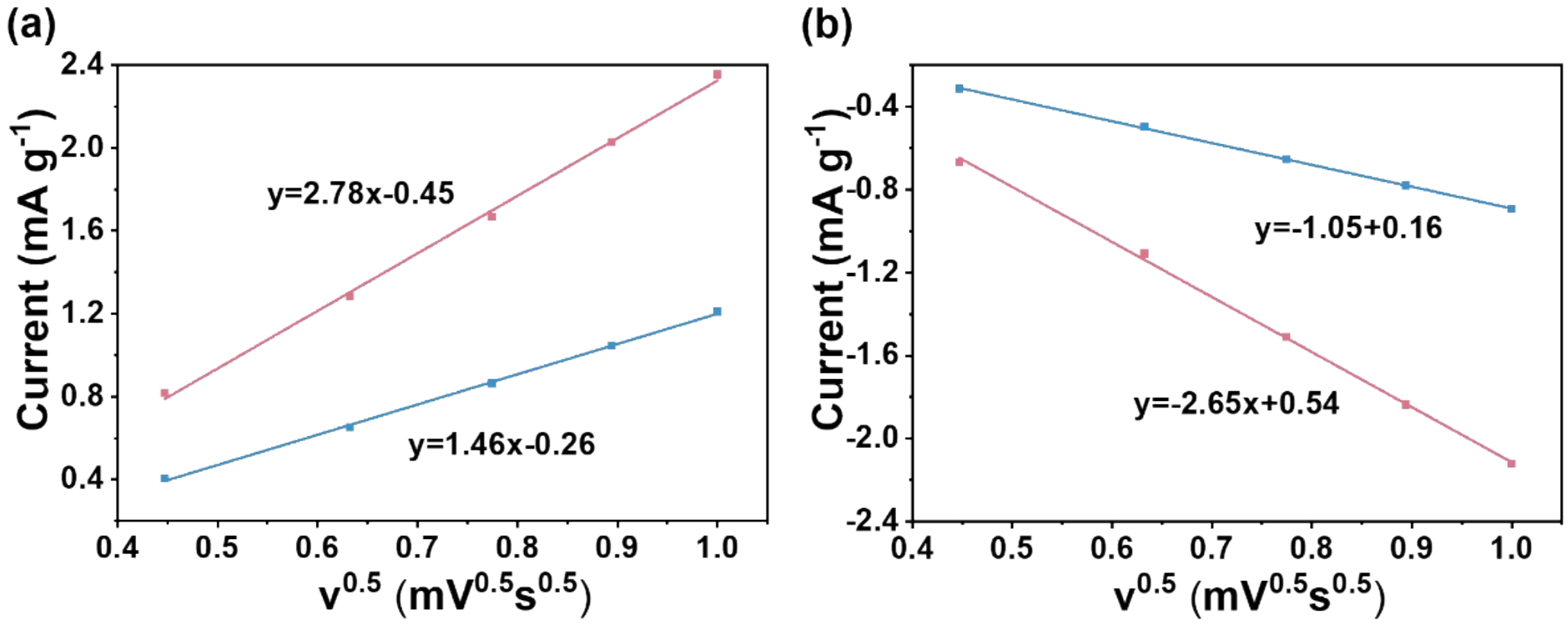


Figure S11. Peak current as a function of the square root of scan rate $v^{0.5}$ extracted from CV curves at various scan rates. (a) Oxidation process. (b) Reduction process.

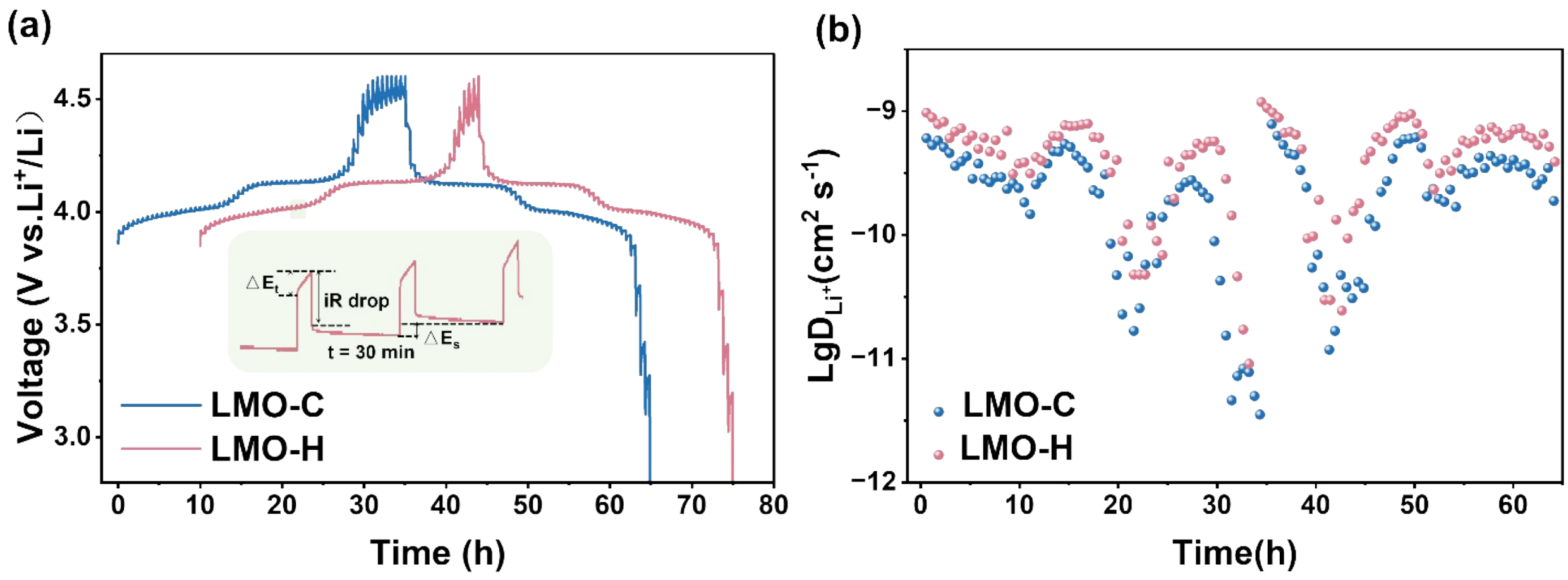


Figure S12. $Li^{+}$ diffusion coefficients of LMO-C and LMO-H battery systems measured via GITT. (a) Voltage-time curves from GITT measurements for LMO-H and LMO-C half-cells after 10 cycles; (b) Lithium-ion diffusion coefficients calculated from the GITT results.

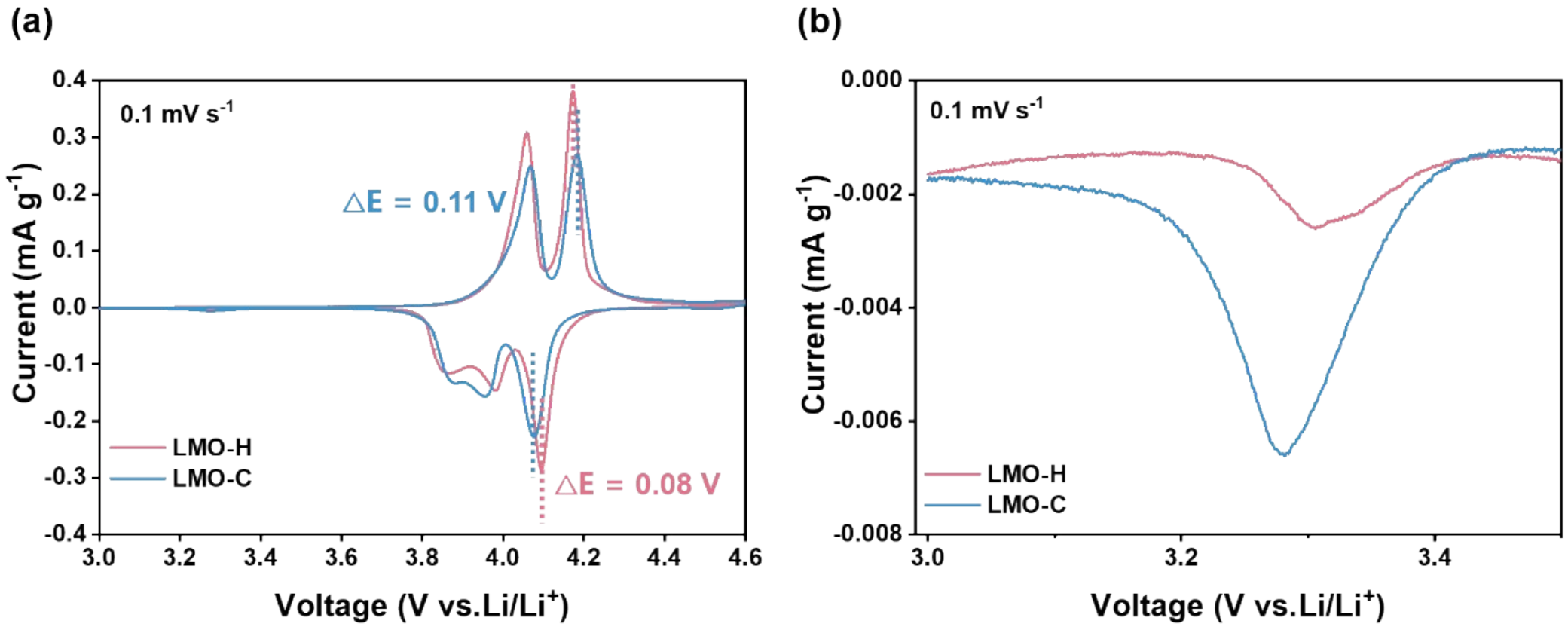


Figure S13. CV curves of LMO-H and LMO-C at a scan rate of 0.1 mV $s^{-1}$. (a) Full curves in the voltage range of 3.0 - 4.6 V. (b) Magnified view of the 3.0 - 3.5 V region.

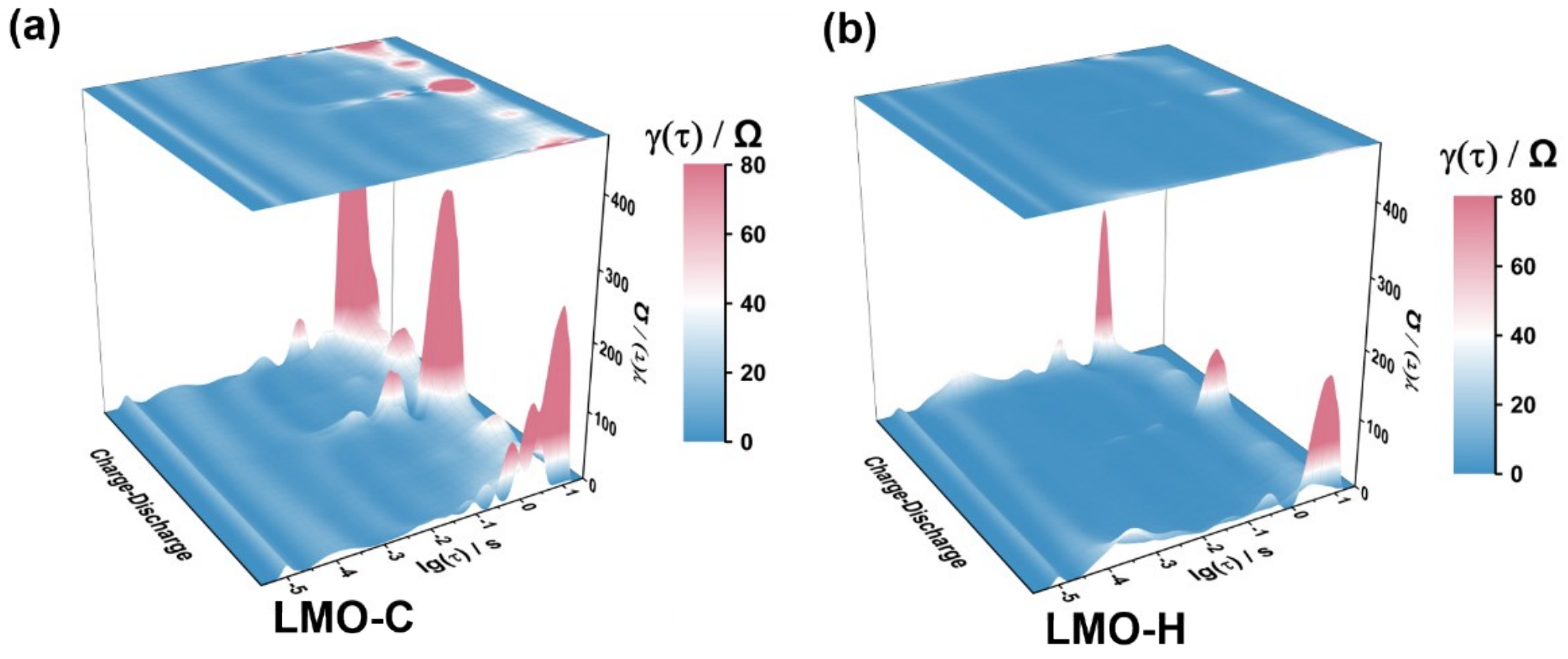


Figure S14. In-situ EIS-DRT images. (a) LMO-C and (b) LMO-H.

Supplementary Table 5. Curve-fit Parameters[a] for Mn K-edge EXAFS for LMO-H.

| Path | N | R/Å | $\sigma^2$/Å$^2$ |
|---|---|---|---|
| Mn-O | 5.4(4) | 1.90(1) | 0.003(1) |
| Mn-Mn | 5.8(2) | 2.91(1) | 0.008(2) |

[a] $S_0^2$ was fixed as 0.9. $\Delta E_0$ was refined as a global fit parameter, returning a value of (-1±1) eV. Data ranges: $2.5 \le k \le 11.5$ Å$^{-1}$,$1.0 \le R \le 3.0$ Å. The number of variable parameters is 7, out of a total of 11.1 independent data points. R factor for this fit is 1.08%.

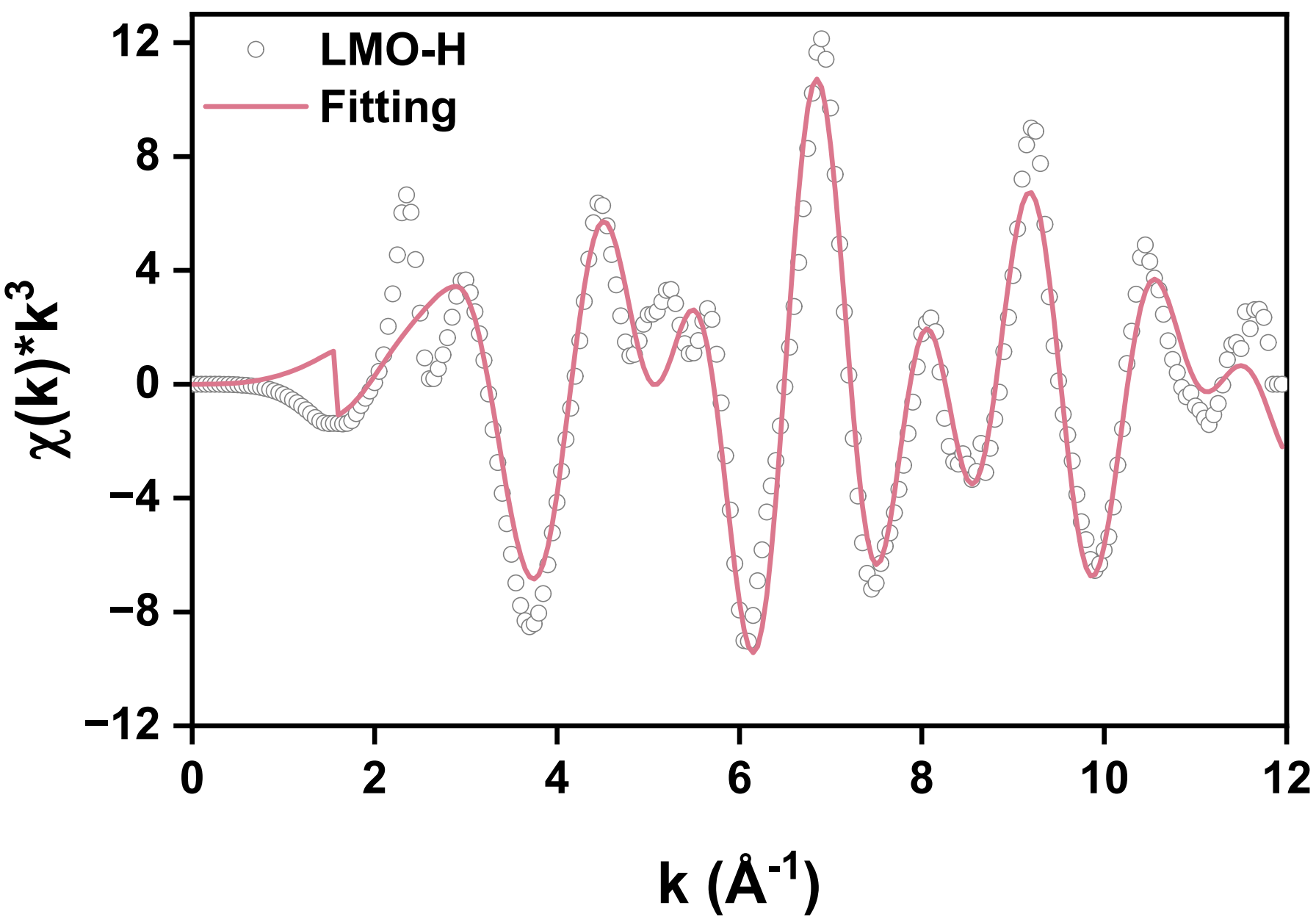


Figure S15. Mn K-edge EXAFS (points) and the curve-fit (line) for LMO-H, shown in $k^3$ weighted k-space.

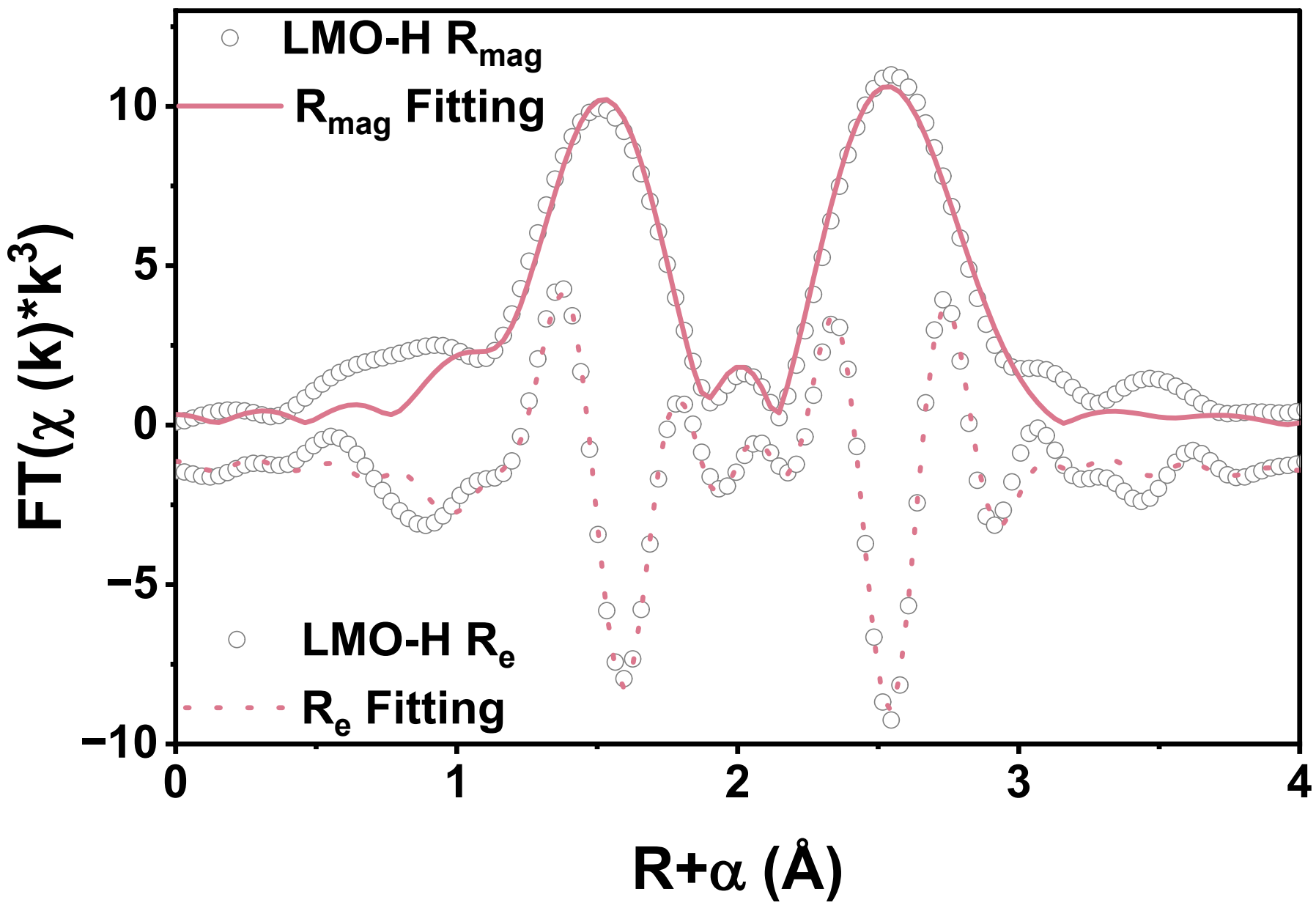


Figure S16. Mn K-edge EXAFS (points) and the curve-fit (line) for LMO-H, shown in R-space (FT magnitude and real component). The data are $k^3$-wighted and not phase-corrected.

Supplementary Table 6. Curve-fit Parameters[a] for Mn K-edge EXAFS for LMO-C.

| Path | N | R/Å | $\sigma^2$/Å$^2$ |
|---|---|---|---|
| Mn-O | 5.1(4) | 1.92(1) | 0.003(1)[c] |
| Mn-Mn | 5.4(5) | 2.90(1) | 0.007(2) |

[a] $S_0^2$ was fixed as 0.9. $\Delta E_0$ was refined as a global fit parameter, returning a value of (-1 ± 1) eV. Data ranges: $2.5 \leq k \leq 11.5$ Å$^{-1}$, $1.0 \leq R \leq 3.0$ Å. The number of variable parameters is 7, out of a total of 11.1 independent data points. R factor for this fit is 1.06%.

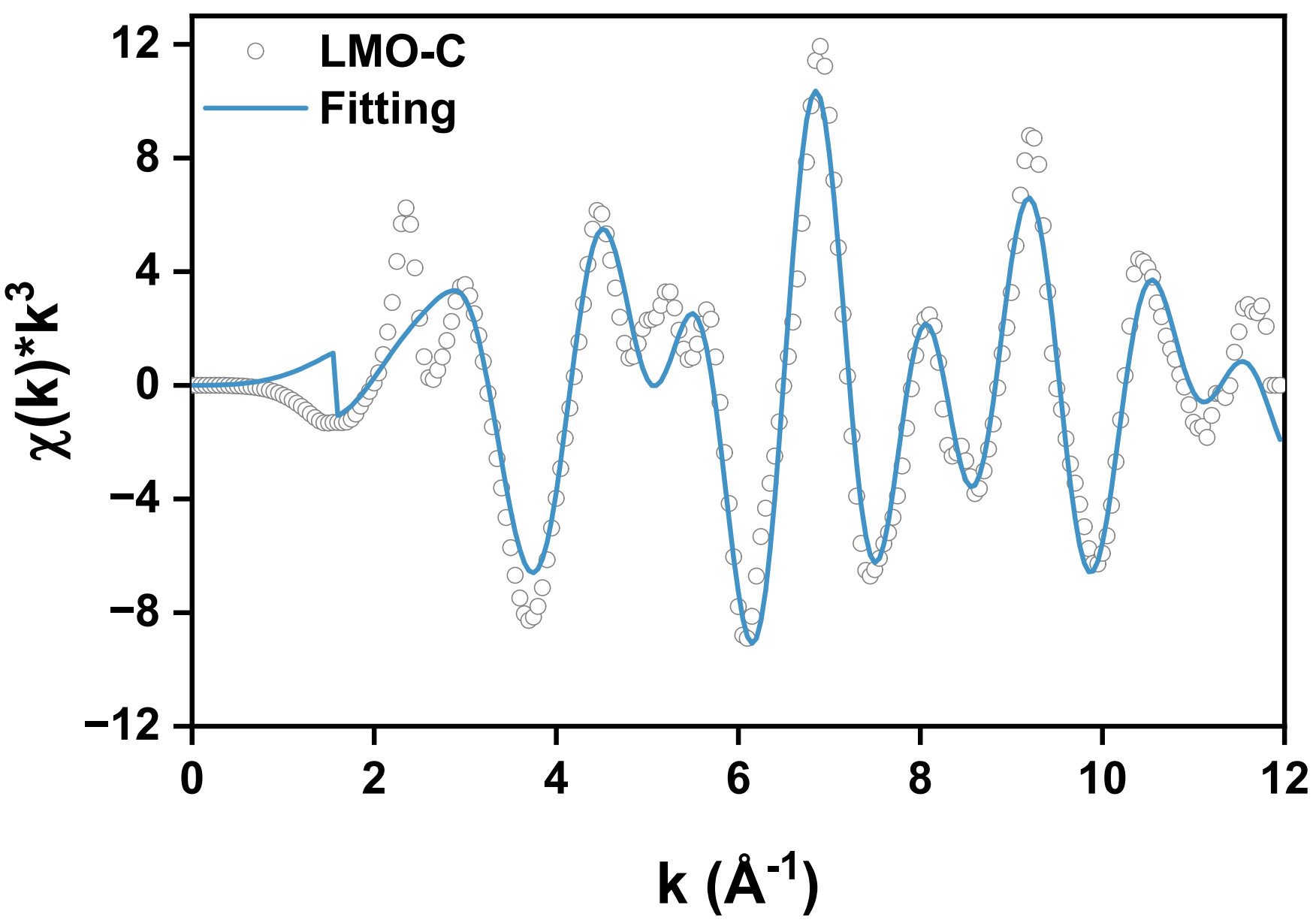


Figure S17. Mn K-edge EXAFS (points) and the curve-fit (line) for LMO-C, shown in $k^3$ weighted k-space.

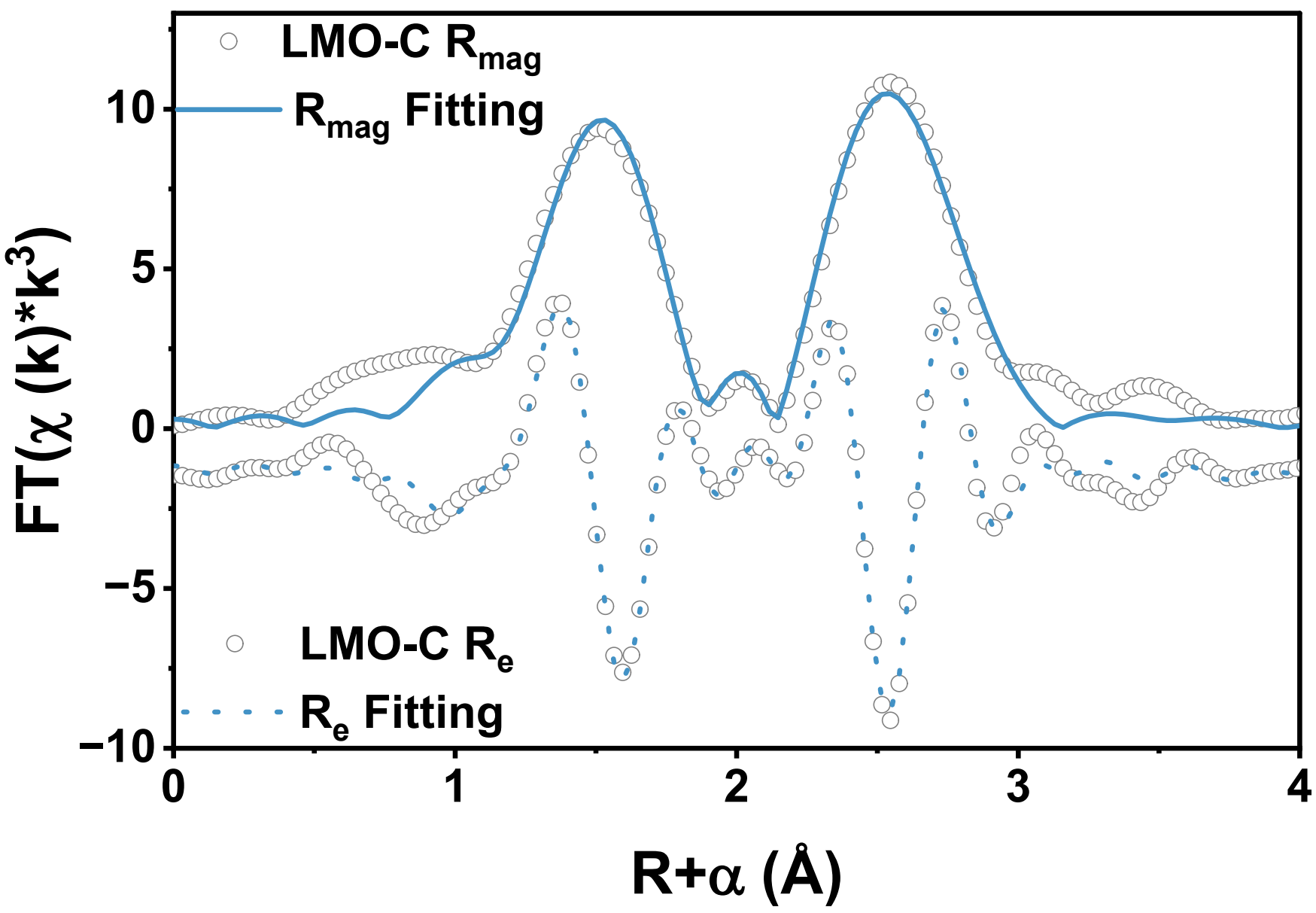


Figure S18. Mn K-edge EXAFS (points) and the curve-fit (line) for LMO-C, shown in R-space (FT magnitude and real component). The data are $k^3$-wighted and not phase-corrected.

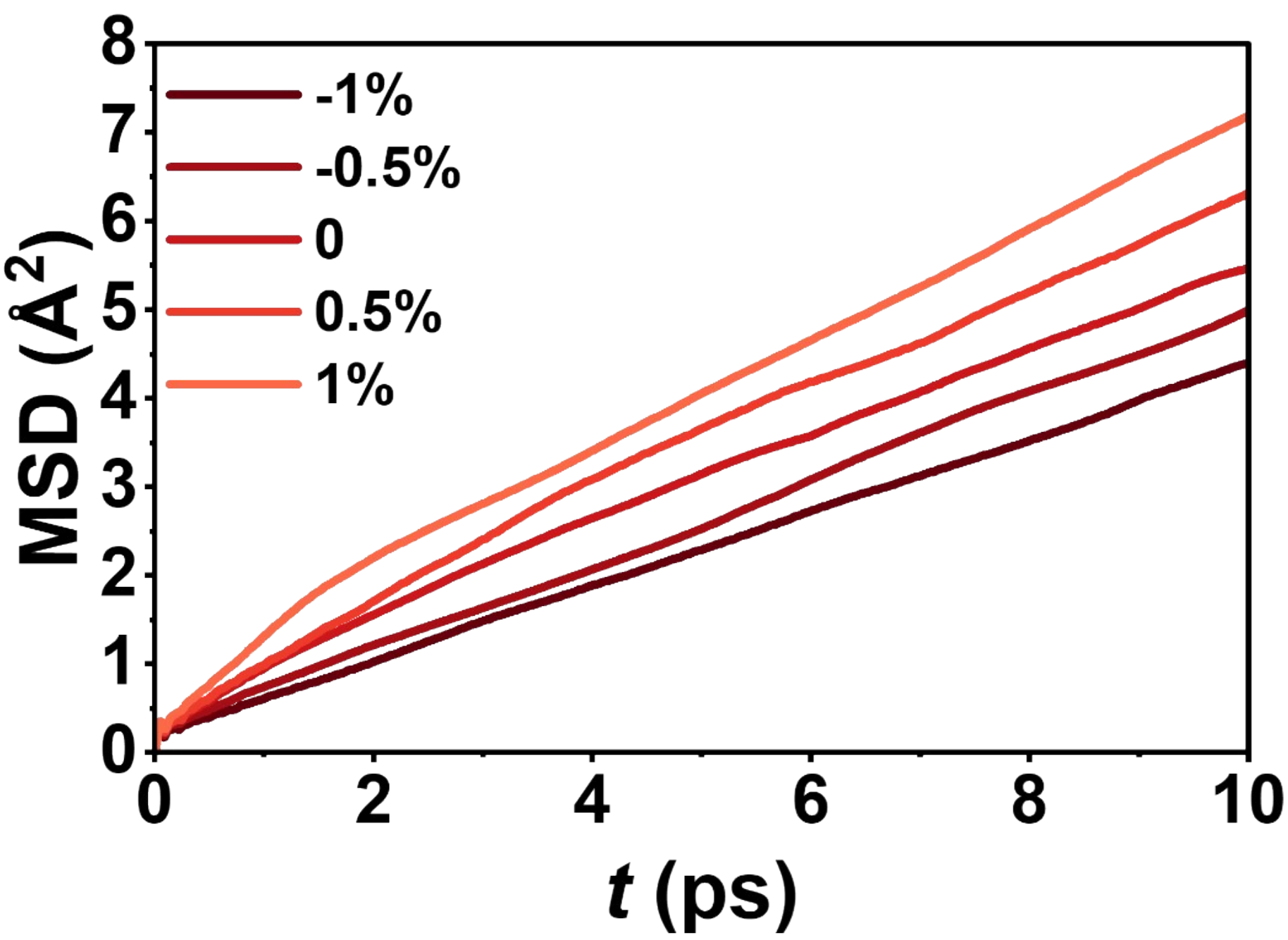


Figure S19. Mean squared displacement (MSD) of $Li^+$ as a function of LMO unit cell parameters.

Supplementary Table 7. Relationship between unit cell parameters and Mn–Mn bond lengths in the DFT model.

| | **-1%** | **-0.5%** | **0** | **+0.5%** | **+1%** |
|---|---|---|---|---|---|
| **a** | 8.26681 | 8.30856 | 8.35031 | 8.39206 | 8.43382 |
| **Mn-Mn** | 2.8995450 | 2.9141892 | 2.9288333 | 2.9434775 | 2.9581217 |

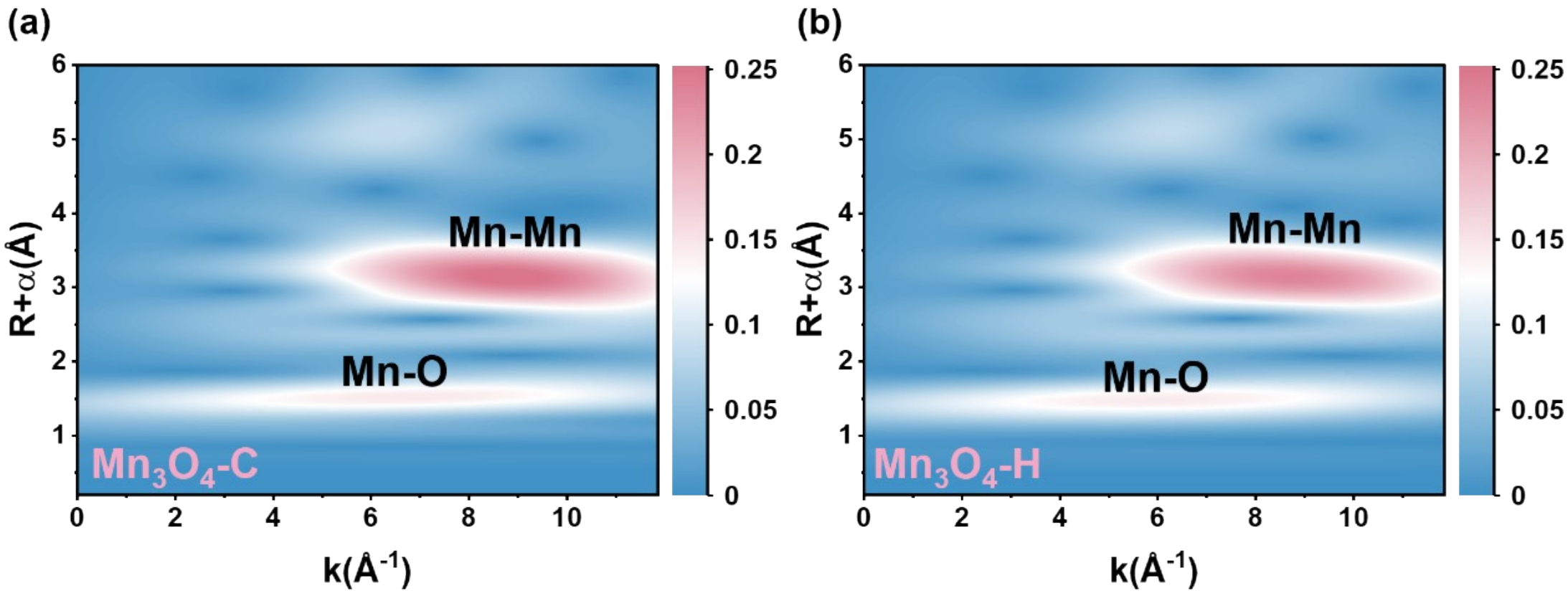


Figure S20. EXAFS wavelet transform images of the different precursors. (a) $Mn_3O_4$-C. (b) $Mn_3O_4$-H.

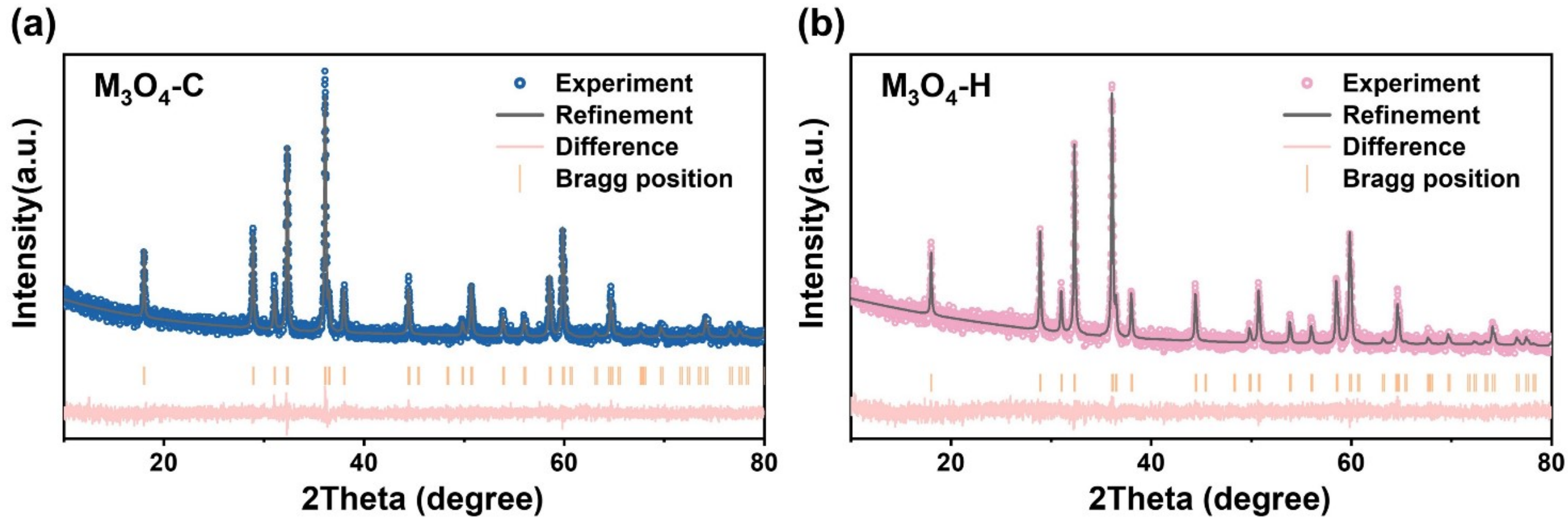


Figure S21. XRD refinement patterns of different precursors. (a) $Mn_3O_4$-C, (b) $Mn_3O_4$-H.

Supplementary Table 8. Lattice parameters of $Mn_3O_4$-C and $Mn_3O_4$-H obtained from XRD refinement.

| **Samples** | **a (Å)** | **b (Å)** | **c (Å)** | **V (Å$^3$)** |
|---|---|---|---|---|
| $M_3O_4$-C | 5.76280 | 5.76280 | 9.47258 | 314.58309 |
| $M_3O_4$-H | 5.76583 | 5.76583 | 9.46529 | 314.67163 |

Supplementary Table 9. Structure parameter obtained from Rietveld refinement of $Mn_3O_4$-C based on a space group of $I4_1$/amd.

| **Name** | **X** | Y | **Z** | **Occ** | **Site** |
|---|---|---|---|---|---|
| Mn1 | 0.00000 | 0.25000 | 0.87500 | 0.12500 | 4a |
| Mn2 | 0.00000 | 0.50000 | 0.50000 | 0.25000 | 8d |
| O | 0.00000 | 0.47220 | 0.25890 | 0.50000 | 16e |

Supplementary Table 10. Structure parameter obtained from Rietveld refinement of $Mn_3O_4$-H based on a space group of $I4_1$/amd.

| **Name** | **X** | Y | **Z** | **Occ** | **Site** |
|---|---|---|---|---|---|
| Mn1 | 0.00000 | 0.25000 | 0.87500 | 0.12259 | 4a |
| Mn2 | 0.00000 | 0.50000 | 0.50000 | 0.24879 | 8d |
| O | 0.00000 | 0.47220 | 0.25890 | 0.50000 | 16e |

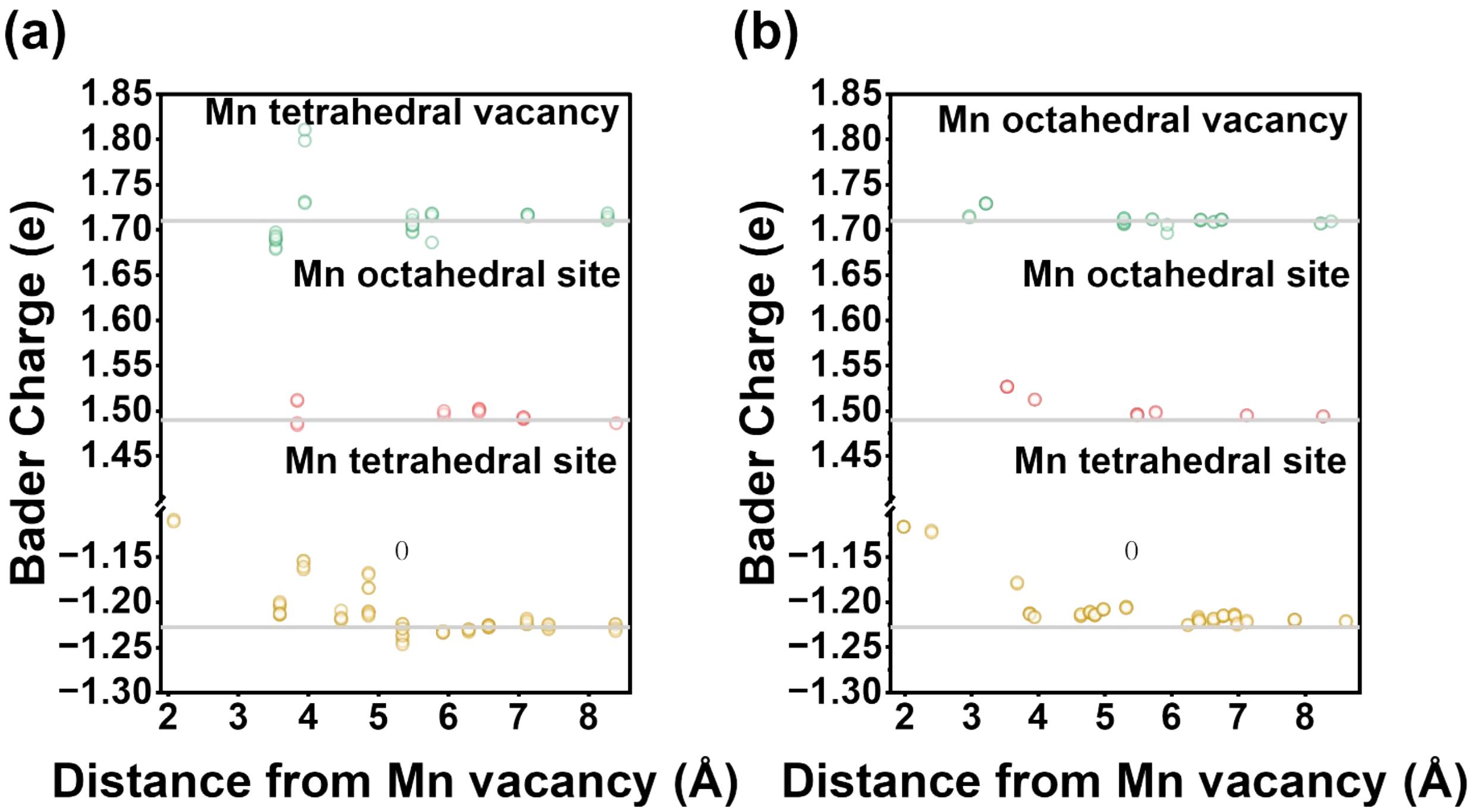


Figure S22. Variation of Bader charges for individual atoms with distance from the defect site under different Mn defect structures (the gray line represents the average Bader charge of $Mn_3O_4$). (a) Mn tetrahedral vacancy, (b) Mn octahedral vacancy.

Supplementary Table 11. Average Bader charges of various types of atoms.

| | $Mn_3O_4$ | Mn tetrahedral vacancy | Mn octahedral vacancy |
|---|---|---|---|
| **Mn octahedral site** | 1.7102 | 1.7120 | 1.7121 |
| **Mn tetrahedral site** | 1.4900 | 1.4964 | 1.5054 |
| **O** | -1.2276 | -1.2067 | -1.2057 |

Supplementary Table 12. Detailed specifications for pouch-cell.

| | | |
|---|---|---|
| Cell capacity (Ah) | 0.40 | |
| Cell weight (g) | 11.17 | |
| Specific energy (Wh $kg^{-1}$) | 141.94 | |
| Power density (kW $kg^{-1}$) | 4.82 (50 C) | |
| Cathode | Active material | $LiMn_2O_4$ (LMO-H) |
| | Active material percentage (%) | 92.00 |
| | Area weight (each side. mg $cm^{-2}$) | 10.00 |
| | Area capacity (each side. mAh $cm^{-2}$) | 1.32 |
| | Length (mm) | 520.00 |
| | Width (mm) | 40.00 |
| | Thickness (two sides, µm) | 68.00 |
| | Aluminum foil thickness (µm) | 13.00 |
| | Compact density (g $cm^{-3}$) | 3.64 |
| Anode | Active material | Graphite |
| | Active material percentage (%) | 95.40 |
| | Area weight (each side. mg $cm^{-2}$) | 4.50 |
| | Length (mm) | 547.00 |
| | Width (mm) | 42.00 |
| | Thickness (two sides, µm) | 79.00 |
| | Copper foil thickness (µm) | 18.00 |
| | Compact density (g $cm^{-3}$) | 1.48 |
| Negative to positive capacity ratio | | 1.10 |
| Thickness of separator (µm) | | 11.00 |
| Thickness of packing foil (µm) | | 111.00 |
| Electrolyte to capacity ratio (g $Ah^{-1}$) | | 5.00 |

**Li-electrode replacement experiment and DRT analysis.**

To further evaluate the contribution of the Li-metal counter electrode to the impedance response of LMO||Li two-electrode cells, a Li-electrode replacement experiment was performed. EIS measurements were first conducted on the cycled LMO||Li cells. The cells were then transferred into an Ar-filled glovebox and carefully disassembled. The cycled Li-metal counter electrodes were removed and replaced with fresh Li metal, while the original separators were retained to minimize changes to the cell configuration and other cell components. A small amount of fresh electrolyte was added to compensate for possible electrolyte loss during cell disassembly and reassembly. The cells were subsequently reassembled and subjected to additional galvanostatic cycling under the same electrochemical conditions as the original cells. EIS measurements were performed after the 1$^{st}$, 2$^{nd}$, 3$^{rd}$, 4$^{th}$, and 5$^{th}$ cycles following Li-electrode replacement, and the corresponding EIS-DRT were analyzed under the same analysis conditions as those applied to the original LMO||Li cells.

The results show that, during the subsequent cycling following replacement with fresh Li, Peak 2 at approximately $10^{-4.5}$ to $10^{-3}$ s and Peak 4 at approximately $10^{-1.5}$ to $10^{-0.5}$ s exhibit the most pronounced changes. With further cycling, the intensities of these two peaks initially decrease and subsequently increase, whereas the other DRT features show comparatively smaller variations. The pronounced sensitivity of Peak 2 and Peak 4 to Li-electrode replacement and their subsequent evolution during cycling indicates that these relaxation processes contain substantial impedance contributions from the Li-metal side.

These results further demonstrate that the DRT response obtained from an LMO||Li two-electrode configuration contains contributions from both the LMO cathode and the Li-metal counter electrode and therefore cannot be attributed exclusively to cathode-side processes. Accordingly, the DRT results

in the main text are discussed in terms of the overall evolution of interfacial reaction and ion-transport resistances within the LMO||Li electrochemical system, rather than assigning the corresponding relaxation features exclusively to cathode-side $R_{SEI}$, $R_{ct}$, or diffusion processes.

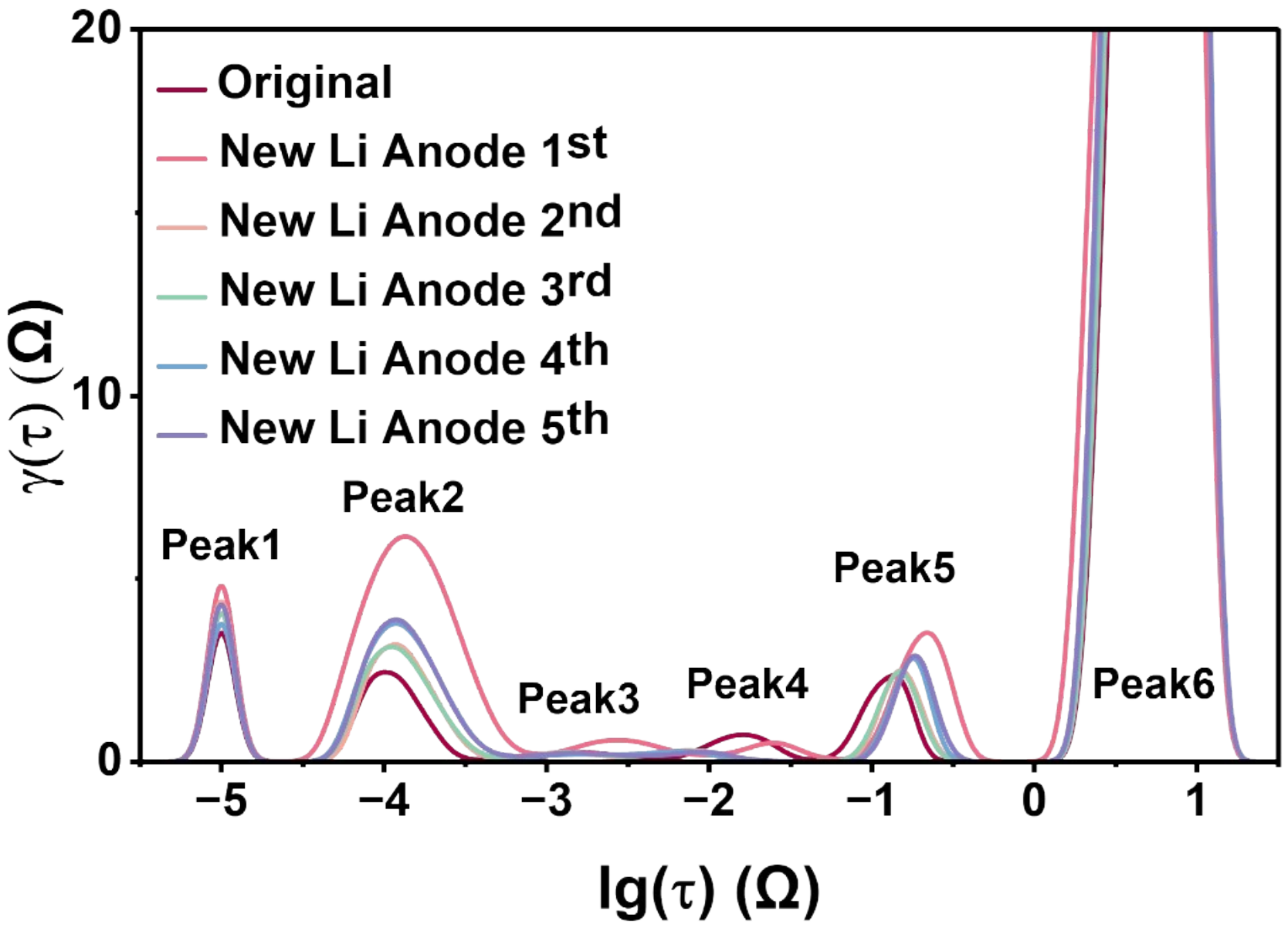


Figure S23. DRT analyses of the LMO||Li cell before and after replacement of the cycled Li-metal counter electrode with fresh Li.

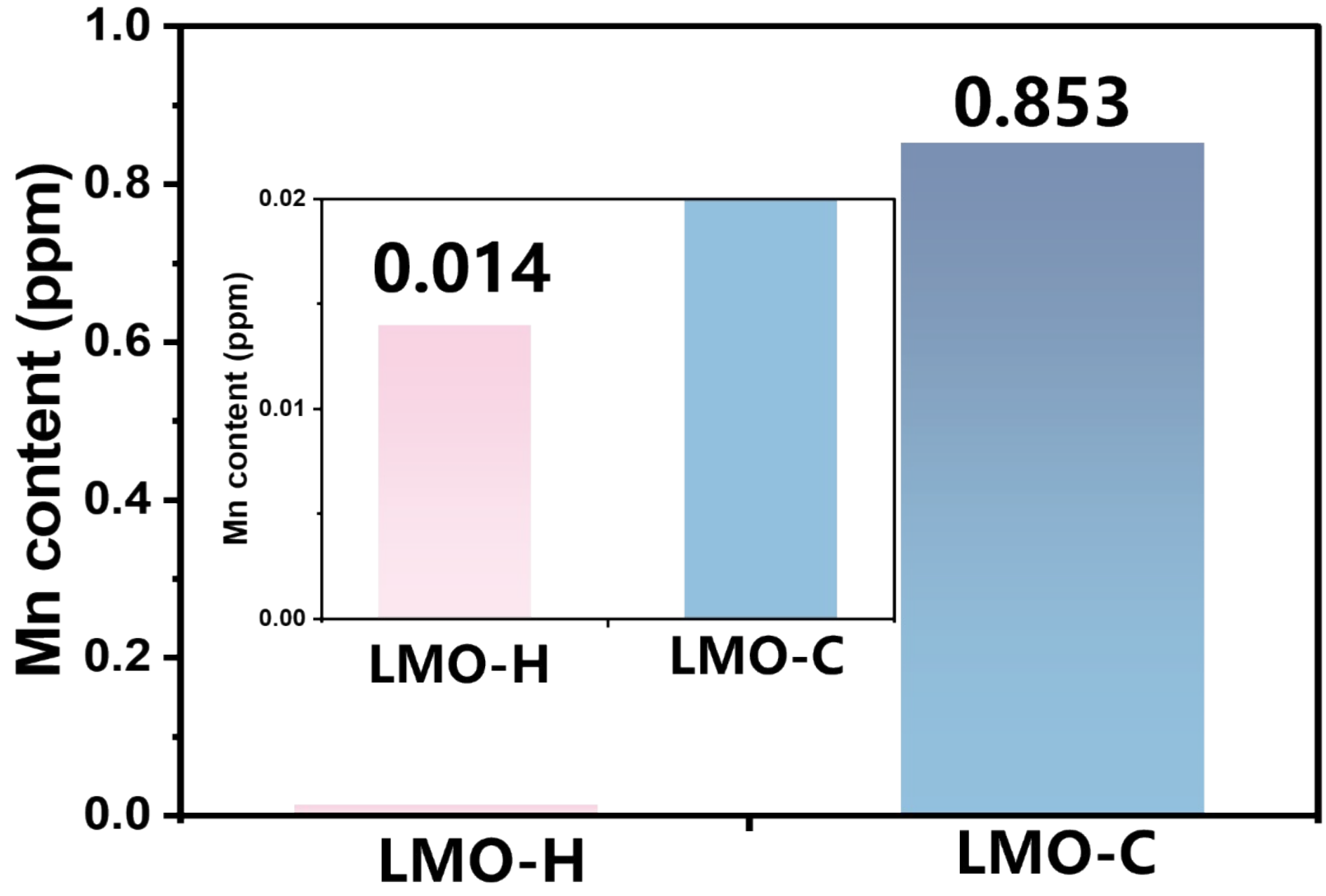


Figure S24. Mn content in lithium anode measured by ICP-OES.

## Cell Assembly and Electrochemical Testing

### Assembly of LMO||Li coin cell

The cathode slurry was prepared by thoroughly mixing $LiMn_2O_4$ (90 wt%), Super P (5 wt%) and poly(vinylidene fluoride) (PVDF, 5 wt%) in N-methyl-2-pyrrolidone (NMP). The resulting slurry was uniformly cast onto carbon-coated aluminium foil and dried under vacuum for 24 h. The dried electrodes were punched into 14 mm diameter disks with an active-material loading of 9-10 mg $cm^{-2}$. LMO||Li half-cells were assembled in CR-2032 coin cells inside an argon-filled glovebox, where the concentrations of $O_2$ and $H_2O$ were maintained below 1 ppm. Each cell consisted of a $LiMn_2O_4$ cathode (14 mm), a glass-fibre separator (18 mm, Whatman GF/A) and a lithium metal foil anode (99.9%, 16 mm, Alfa Aesar). Additionally, ethylene carbonate (EC), diethyl carbonate (DEC) at a ratio of 1:1 (vol%) consisting of 1 M $LiPF_6$ and 10% FEC was used as the electrolyte. The electrolyte amount is 60 μL, and the Cells were rested for 12 h before electrochemical testing.

### Electrochemical performance characterization

Then, the galvanostatic charge/discharge tests were carried out on the Neware battery testing system (CT-4008T-5V100mA-164) between 3.2 V and 4.6 V at different C-rates (1 C-30 C, 1 C = 148 mA $g^{-1}$). Cyclic voltammetry (CV) tests were performed on an electrochemical workstation (PARSTAT 3000A-DX, Princeton Applied Research, USA) at a scan rate of 0.2 to 1 mV $s^{-1}$. The ex-situ electrochemical impedance spectroscopy data of cycled batteries were carried out on the cathodes at the charged state of 4.6 V at frequencies from 0.1 Hz to $1\times10^5$ Hz with a sinusoidal excitation voltage of 10 mV. The in-situ electrochemical impedance spectroscopy (EIS) measurements were performed using a dual-channel electrochemical workstation (PARSTAT 3000A-DX, Princeton Applied Research, USA), with one channel used for galvanostatic charge–discharge cycling and the other for

simultaneous impedance measurements. The DRT analyses were performed using the MATLAB GUI toolbox designed by Ciucci's research group. [4, 5] Galvanostatic intermittent titration technique (GITT) measurements were performed using a Neware battery testing system (CT-4008T-5V100mA-164) during the $10^{th}$ cycle over a voltage range of 3.0 -4.6 V. A constant-current pulse of 0.2 C was applied for 5 min, followed by an open-circuit relaxation period of 30 min before the subsequent pulse. The apparent $Li^+$ diffusion coefficient ($D_{Li^+}$) was calculated according to the following equation:

$$D_{Li^+} = \frac{4}{\pi\tau}\left(\frac{m_B V_m}{M_B S}\right)^2\left(\frac{\Delta E_s}{\Delta E_t}\right)^2$$

where τ is the duration of the current pulse; $m_B$ and $M_B$ are the mass and molar mass of the active material, respectively; $V_m$ is the molar volume of LMO; and S is the geometric area of the active electrode. $\Delta E_t$ represents the change in cell voltage during the constant-current pulse, excluding the instantaneous ohmic (iR) drop, whereas $\Delta E_s$ represents the change in the steady-state voltage between two successive relaxation steps.

**Assembly of LMO||graphite pouch-cell**

**Cathode preparation.**

The cathode slurry was prepared by mixing LMO-H (92.0 wt%), carbon nanotubes (1.0 wt%), Super P (2.5 wt%) and poly(vinylidene fluoride) (4.5 wt%, PVDF) in N-methyl-2-pyrrolidone (NMP). Specifically, PVDF was first dissolved in NMP under stirring for 3 h, followed by the addition of CNTs and Super P and further stirring for 4 h. LMO-H was then added, and the mixture was stirred for another 4 h to obtain a homogeneous slurry. The slurry was subsequently filtered through a 200-mesh sieve, coated onto Al foil and dried under vacuum at 180 °C for 24 h. After calendaring, the thickness of the double-side-coated cathode was 68 μm.

**Anode preparation.**

The anode slurry was prepared by mixing high-rate graphite (95.4 wt%, BTR), Super P (1.0 wt%), styrene–butadiene rubber (2.5 wt%, SBR) and sodium carboxymethyl cellulose (1.1 wt%, CMC) in deionized water. The resulting slurry was coated onto Cu foil and dried under vacuum at 180 °C for 24 h. After calendaring, the thickness of the double-side-coated anode was 79 μm.

**Pouch-cell assembly and testing.**

The calendared cathode and anode electrodes were punched into designed dimensions and assembled into pouch cells by winding, tab welding and sealing in aluminium-laminated pouches. The assembled cells were dried at 85 °C for 48 h to ensure a total moisture content below 70 ppm. The cells were then filled with electrolyte, using the same electrolyte formulation as that used for the coin cells, with an electrolyte consumption of approximately 5 g $Ah^{-1}$, and rested at 45 °C for 36 h. Formation was carried out at 45 °C by charging at 0.2 C for 30 min, followed by charging at 0.5 C for 60 min and then at 0.1 C to 4.25 V. After formation, the cells were degassed and resealed. All pouch-cell electrochemical tests were conducted using a Neware MGDW-225-40H battery testing system at 25 °C within a voltage window of 3.0-4.25 V.

**Materials characterizations**

**X-ray diffraction (XRD)：**

The crystal structure of the synthesized materials were characterized using XRD (Rigaku, SmartLab SE, Japan) with Cu Kα radiation, and the Rietveld refinement was performed using Fullprof.[6]

**In situ high-temperature XRD:**

The phase evolution during lithiation was monitored by in situ high-temperature XRD. Stoichiometric mixtures of the $Mn_3O_4$ precursor and $Li_2CO_3$ were loaded onto a heating stage and heated in an oxygen atmosphere at a heating rate of 5 °C $min^{-1}$. Diffraction patterns were collected at 20 °C intervals over a 2θ range of 10°-80° with a scanning rate of 10° $min^{-1}$.

**Scanning electron microscopy (SEM)：**

The morphology of the materials were examined by SEM (Hitachi, SU8200, Japan).

**Transmission electron microscope (TEM):**

The morphology and microstructure of the samples were observed using a JEM-F200 TEM (JEOL) with a 200 kV acceleration voltage high-resolution transmission electron microscope (HR-TEM).

**Inductively coupled plasma optical emission spectrometry (ICP-OES)：**

Quantification of Mn dissolution: After 500 charge-discharge cycles, the coin cells were disassembled using an MSK-110 battery disassembly device (Kejing). The lithium metal anodes were retrieved, repeatedly rinsed with diethyl carbonate (DEC) to remove residual electrolyte, and dried in a vacuum transfer chamber. The lithium metal was completely dissolved in deionized water. The resulting solution was acidified with 0.2 M $HNO_3$ and diluted to a final volume of 50 mL. The Mn concentration in the solution was quantified by ICP-OES (5100 SVDV, Agilent, USA) to evaluate the extent of Mn dissolution from the cathode during electrochemical cycling. For quantitative elemental

analysis, three independently weighed samples of dried $Mn_3O_4$-H and $Mn_3O_4$-C were completely dissolved in a mixture of HCl and $HNO_3$ (3:1, v/v). After complete dissolution and clarification of the solutions, each solution was diluted to a final volume of 100 mL. Subsequently, a 1 mL aliquot of each solution was further diluted to 25 mL with deionized water and analysed by ICP-OES to determine the Mn content. Similarly, three independently weighed samples of the calcined LMO-H, LMO-H-800, LMO-C, and LMO-C-Bead Milling materials were completely dissolved in a mixture of HCl and $HNO_3$ (3:1, v/v), followed by dilution to a final volume of 100 mL. A 5 mL aliquot of each solution was then further diluted to 25 mL with deionized water and subjected to ICP-OES analysis. The Li and Mn concentrations were quantified, and the corresponding Li/Mn molar ratios were calculated accordingly.

**Synchrotron-based X-ray absorption fine structure (XAFS):**

XAFS measurements were carried out at room temperature at the BL17B1 beamline of the National Facility for Protein Science in Shanghai (NFPS) at the Shanghai Synchrotron Radiation Facility (SSRF). Mn K-edge X-ray absorption spectra were collected in transmission mode. The acquired XAFS data were processed using the Athena and Artemis programs within the IFEFFIT package. [7, 8] Specifically, the X-ray absorption near-edge structure (XANES) spectra were background-corrected, normalized, and energy-calibrated against standard reference materials in Athena. The extended X-ray absorption fine structure (EXAFS) signals were further converted into k-space and weighted by $k^3$ to compensate for amplitude reduction at high k values. Quantitative structural refinements were subsequently performed in Artemis using $\chi(k)\cdot k^3$ data after Fourier transformation into R space.

**First-Principles Calculation Method**

The solid-state transformation from $Mn_3O_4$ to $LiMn_2O_4$ requires progressive incorporation of Li into the $Mn_3O_4$ lattice together with sufficient mass transport. We therefore compared the thermodynamic energy changes associated with Li incorporation into Mn-vacancy-containing and pristine $Mn_3O_4$ models to evaluate the effect of pre-existing Mn vacancies on the Li-incorporation process.

All first-principles calculations were performed within the framework of density functional theory (DFT) as implemented in the Vienna Ab initio Simulation Package (VASP).[8] Exchange and correlation were treated within the generalized gradient approximation (GGA) using the Perdew-Burke-Ernzerhof (PBE) functional.[9] The interaction between core and valence electrons was described by the projector augmented-wave (PAW) method,[9, 10] and the Kohn-Sham orbitals were expanded in a plane-wave basis set with a kinetic energy cutoff of 520 eV. The valence electron configurations for Li, Mn, and O were explicitly treated as $1s^2\ 2s^1$, $3p^6\ 3d^5\ 4s^2$, and $2s^2\ 2p^4$, respectively. All calculations were spin-polarized. To correct the self-interaction error associated with the localized Mn 3d electrons, the rotationally invariant DFT+$U$ scheme of Dudarev et al. was applied to the Mn 3d states with an effective Hubbard parameter $U_{eff} = 4.84$ eV.[12] Long-range dispersion was included through Grimme's D3 correction with Becke–Johnson damping (DFT-D3(BJ)).[13] For structural relaxations, the Brillouin zone was sampled using a Monkhorst-Pack 3 × 3 × 3 k-point grid. Ionic positions and cell parameters were relaxed until all residual Hellmann–Feynman forces were below 0.01 eV $Å^{-1}$.

The defect models were constructed from a 2 × 2 × 1 supercell of tetragonal $Mn_3O_4$ (hausmannite, space group $I4_1/amd$, No. 141) built from the conventional cell. Three structural models were considered: (i) pristine $Mn_3O_4$; (ii) $Mn_3O_4$ containing a single vacancy on a tetrahedrally coordinated Mn site ($V_{Mn}$); and (iii) Li-substituted $Mn_3O_4$ in which one Li occupies the same tetrahedral site ($Li_{Mn}$). To examine the preferred crystallographic site of the Mn vacancy, an additional $Mn_3O_4$ model

containing a single vacancy on an octahedrally coordinated Mn site was constructed. The formation energy of an Mn vacancy was calculated as

$$\Delta Evac = E(Mn_3O_4 : V_{Mn}) - E(Mn_3O_4) + \mu_{Mn} \qquad (1)$$

The energy of filling a pre-existing tetrahedral Mn vacancy with Li was defined as

$$\Delta E_{fill} = E(Mn_3O_4 : Li_{Mn}) - E(Mn_3O_4 : V_{Mn}) - \mu_{Li} \qquad (2)$$

whereas the energy of substituting Li for Mn relative to pristine $Mn_3O_4$ was defined as

$$\Delta E_{sub} = E(Mn_3O_4 : Li_{Mn}) + \mu_{Mn} - E(Mn_3O_4) - \mu_{Li} \qquad (3)$$

Here $E$ denotes the total energy of the corresponding fully relaxed supercell, $\mu_{Li}$ and $\mu_{Mn}$ are the chemical potentials of Li and Mn. The chemical potentials were referenced to the elemental bulk metals.

To investigate the effect of lattice expansion and the associated Mn-Mn distance on $Li^+$ migration in spinel $LiMn_2O_4$, ab initio molecular dynamics (AIMD) simulations were performed using $Li_7Mn_{16}O_{32}$ models containing one Li vacancy. The DFT-optimized lattice parameter was taken as reference, and five models were constructed with lattice parameters scaled by factors of 0.990, 0.995, 1.000, 1.005, and 1.010, respectively. AIMD simulations were carried out in the canonical (NVT) ensemble at 1000 K using a time step of 1 fs, with the Brillouin zone sampled at the Γ point. The $Li^+$ mean-square displacement (MSD) was calculated from the AIMD trajectories to evaluate $Li^+$ migration, and the average Mn-Mn distances were analyzed for the corresponding lattice-scaled structures.

## Reference


1. Lee, G. J.; Abbas, M. A.; Lee, M. D.; Lee, J.; Lee, J.; Bang, J. H., Lithiation mechanism change driven by thermally induced grain fining and its impact on the performance of $LiMn_2O_4$ in lithium-ion batteries. *Small* 2020, **16** (29), 2002292.
2. Pomerantsev, A. L.; Kutsenova, A. V.; Rodionova, O. Y., Kinetic analysis of non-isothermal solid-state reactions: multi-stage modeling without assumptions in the reaction mechanism. *Physical Chemistry Chemical Physics* 2017, **19** (5), 3606-3615.
3. Khawam, A.; Flanagan, D. R., Solid-state kinetic models: Basics and mathematical fundamentals. *ChemInform* 2006, **37** (47).
4. Wang, Z.; Wang, Y.; Py, B.; Maradesa, A.; Liu, J.; Wan, T. H.; Saccoccio, M.; Ciucci, F., DRTtools: Freely accessible distribution of relaxation times analysis for electrochemical impedance spectroscopy. *ACS Electrochemistry* 2025, **1** (12), 2680-2689.
5. Wan, T. H.; Saccoccio, M.; Chen, C.; Ciucci, F., Influence of the discretization methods on the distribution of relaxation times deconvolution: Implementing radial basis functions with DRTtools. *Electrochimica Acta* 2015, **184**, 483-499.
6. Rodríguez-Carvajal, J., Recent advances in magnetic structure determination by neutron powder diffraction. *Physica B: Condensed Matter* 1993, **192** (1), 55-69.
7. Newville, M., IFEFFIT: interactive XAFS analysis and FEFF fitting. *Journal of Synchrotron Radiation* 2001, **8** (2), 322-324.
8. Kresse, G.; Furthmüller, J., Efficiency of ab-initio total energy calculations for metals and semiconductors using a plane-wave basis set. *Computational Materials Science* 1996, **6** (1), 15-50.
9. Perdew, J. P.; Burke, K.; Ernzerhof, M., Generalized gradient approximation made simple. *Physical Review Letters* 1996, **77** (18), 3865-3868.
10. Blöchl, P. E., Projector augmented-wave method. *Physical Review B* 1994, **50** (24), 17953-17979.
11. Kresse, G.; Joubert, D., From ultrasoft pseudopotentials to the projector augmented-wave method. *Physical Review B* 1999, **59** (3), 1758-1775.
12. Xu, B.; Meng, S., Factors affecting Li mobility in spinel $LiMn_2O_4$—A first-principles study by GGA and GGA+U methods. *Journal of Power Sources* 2010, **195** (15), 4971-4976.
13. Grimme, S.; Ehrlich, S.; Goerigk, L., Effect of the damping function in dispersion corrected density functional theory. *Journal of Computational Chemistry* 2011, **32** (7), 1456-1465.
